# Wnt-PLC-IP_3_-Connexin-Ca^2+^ axis maintains ependymal motile cilia in zebrafish spinal cord

**DOI:** 10.1038/s41467-020-15248-2

**Published:** 2020-04-20

**Authors:** Jun Zhang, Gopalakrishnan Chandrasekaran, Wenting Li, Dong-Young Kim, In Young Jeong, So-Hyun Lee, Ting Liang, Jin Young Bae, Isaac Choi, Hyuno Kang, Jin-Soo Maeng, Myeong-Kyu Kim, Taewon Lee, Seung Woo Park, Min Jung Kim, Hyung-Seok Kim, Hyunju Ro, Yong Chul Bae, Hae-Chul Park, Eun Young Choi, Seok-Yong Choi

**Affiliations:** 1 Department of Biomedical Sciences, Chonnam National University Medical School, Hwasun, 58128 Republic of Korea; 2 Center for Creative Biomedical Scientists at Chonnam National University, Hwasun, 58128 Republic of Korea; 3 Department of Molecular Medicine, Graduate School, Chonnam National University, Hwasun, 58128 Republic of Korea; 4 Department of Biomedical Sciences, University of Ulsan College of Medicine, Seoul, 05505 Republic of Korea; 5 Department of Biomedical Sciences, Korea University, Ansan, 15355 Republic of Korea; 6 Department of Anatomy and Neurobiology, School of Dentistry, Kyungpook National University, Daegu, 41940 Republic of Korea; 7 Department of Biological Sciences, College of Bioscience and Biotechnology, Chungnam National University, Daejeon, 34134 Republic of Korea; 8 Division of Analytical Science, Korea Basic Science Institute, Daejeon, 34133 Republic of Korea; 9 Research Group of Bioprocess Engineering, Korea Food Research Institute, Wanju-gun, 55365 Republic of Korea; 10 Center for Convergent Research of Emerging Virus Infection, Korea Institute of Chemical Technology, Daejeon, 34114 Republic of Korea; 11 Department of Neurology, Chonnam National University Medical School, Gwangju, 61469 Republic of Korea; 12 Division of Applied Mathematical Sciences, College of Science and Technology, Korea University, Sejong, 30019 Republic of Korea; 13 Department of Internal Medicine, Yonsei University College of Medicine, Seoul, 03722 Republic of Korea; 14 Department of Biological Sciences, Sookmyung Women’s University, Seoul, 04310 Republic of Korea; 15 Department of Forensic Medicine, Chonnam National University Medical School, Hwasun, 58128 Republic of Korea

**Keywords:** Ciliogenesis, Differentiation

## Abstract

Ependymal cells (ECs) are multiciliated neuroepithelial cells that line the ventricles of the brain and the central canal of the spinal cord (SC). How ependymal motile cilia are maintained remains largely unexplored. Here we show that zebrafish embryos deficient in Wnt signaling have defective motile cilia, yet harbor intact basal bodies. With respect to maintenance of ependymal motile cilia, *plcδ3a* is a target gene of Wnt signaling. Lack of Connexin43 (Cx43), especially its channel function, decreases motile cilia and intercellular Ca^2+^ wave (ICW) propagation. Genetic ablation of *cx43* in zebrafish and mice diminished motile cilia. Finally, *Cx43* is also expressed in ECs of the human SC. Taken together, our findings indicate that gap junction mediated ICWs play an important role in the maintenance of ependymal motile cilia, and suggest that the enhancement of functional gap junctions by pharmacological or genetic manipulations may be adopted to ameliorate motile ciliopathy.

## Abstract (web summary)

Ependymal cells are supporting cells in the central nervous system. Here the authors elucidate a signalling axis in zebrafish spinal cord ependymal cells that is important for motile cilia assembly and maintenance, demonstrating that it depends on intercellular propagation of calcium ions via connexin 43.

## Introduction

Cilia are a highly conserved organelle that projects from the cell body and classified into motile and nonmotile cilia (also called sensory or primary cilia). Motile cilia move the overlying fluid by coordinated beating and are found in ECs, respiratory epithelial cells, oviduct cells, and node cells. Dysfunctional motile cilia can cause primary ciliary dyskinesia that features chronic recurrent respiratory infections, hydrocephalus, laterality defect, and infertility^1–3^.

Motile ciliated cells have been reported to play an important role in the neurodevelopment of the vertebrates including zebrafish. Knockdown of genes implicated in intraflagellar transport (IFT) impairs flow of the cerebrospinal fluid (CSF) in zebrafish^4^. Motile cilia in the zebrafish otic vesicles are required for proper otolith formation^5^. The CSF flow generated by motile cilia in the zebrafish ventricles is crucial in ventricular development^6^.

Several reports have provided insights into the molecular mechanism underlying EC development. First, adult ECs in the mouse SC are derived from Nkx6.1 expressing ventral neural progenitor cells^7^. Second, ECs are derived from radial glial cells in the developing mouse forebrain^8^. Third, the homeobox gene *Six3* is required for the maturation of brain ECs^9^. Fourth, Sonic hedgehog (Shh) signaling is required for the development of ECs in the developing mouse SC^10^. ECs feature motile cilia on the apical surface and zonula adherens on the lateral surface, and the coordinated beating of these motile cilia circulates the CSF^11^. However, the molecular mechanism underlying the maintenance of motile cilia in ECs remains unclear. Hence, we set out to determine the molecular mechanism using zebrafish as a primary model organism.

Our findings show that the Wnt-PLC-IP_3_-Connexin-Ca^2+^ axis is very likely to be required for the maintenance of the ependymal motile cilia in the zebrafish SC.

## Results

### Wnt signaling is involved in the maintenance of ependymal motile cilia in zebrafish embryos

**Fig. 1:**
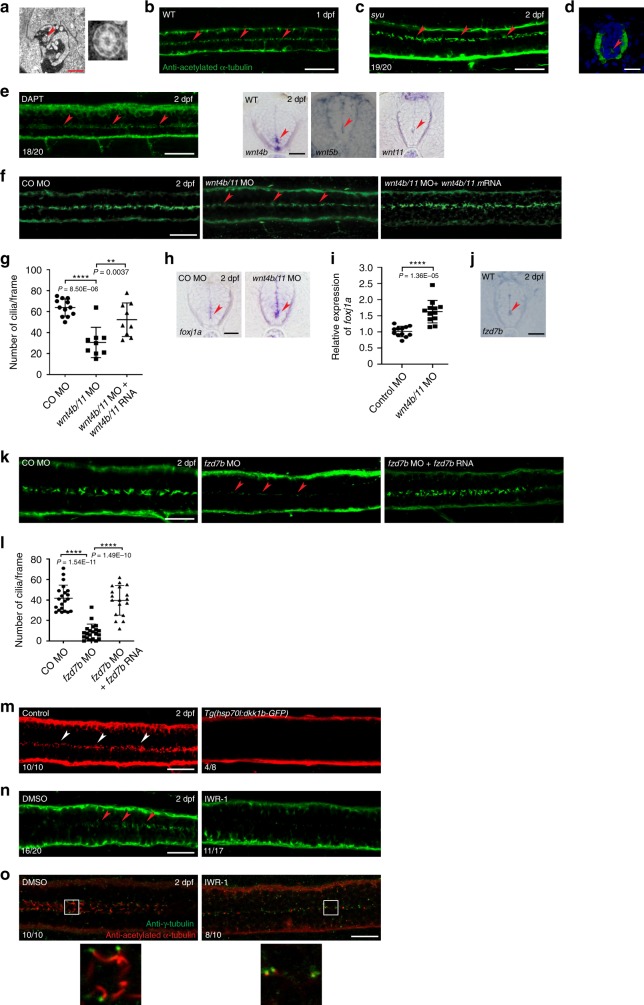
Wnt signaling is involved in the maintenance of ependymal motile cilia in zebrafish embryos. **a** Transmission electron microscopy (TEM) of the spinal cords (SCs) of zebrafish embryos at 2 dpf. Arrowhead indicates a motile cilium with the 9 + 2 microtubule configuration, which is magnified to the right. Scale bar = 1 μm. **b** Immunofluorescence (IF) staining of an embryo at 1 dpf with anti-acetylated-α-tubulin antibody. Dorsal view anterior to the left. Arrowheads represent motile cilia. Scale bar = 20 μm. **c**, **d** IF staining of *sonic-you* (*syu*^*t4*^) mutant embryos at 2 dpf with anti-acetylated-α-tubulin antibody. Dorsal view anterior to the left (**c**). **d** The cross-section image of the SC ventral to the bottom. Arrowheads represent motile cilia. Scale bars = 20 μm. **e** Embryos were treated with DAPT (50 μM) at 34–48 hpf and IF stained with anti-acetylated-α-tubulin antibody. Dorsal view anterior to the left. Arrowheads represent motile cilia. Scale bar = 20 μm. **f** Embryos were co-microinjected with *wnt4b* MO and *wnt11* MO (*wnt4b/11* MO) alone or along with *wnt4b* mRNA and *wnt11* mRNA (*wnt4b/11* mRNA), and IF stained at 2 dpf with anti-acetylated-α-tubulin antibody. Arrowheads represent motile cilia. Dorsal view anterior to the left. Scale bar = 20 μm. CO: Control. **g** Quantification of the number of cilia per frame in embryos in **f**. Data are presented as mean ± SD. **P < 0.01 and ****P < 0.0001 by one-way ANOVA with Tukey’s honest significant difference (HSD) post hoc test (control morphants: *n* = 12 embryos; *wnt4b/11* double morphants: *n* = 9 embryos; *wnt4b/11* double morphants + *wnt4b/11* mRNA: *n* = 9 embryos; one frame per embryo). **h** A cross-section images of the SCs of control morphants and *wnt4b/11* double morphants at 2 dpf probed with *foxj1a* riboprobes ventral to the bottom. Arrowheads represent ECs. Scale bar = 20 μm. CO: Control. **i** RNAs were extracted from each group (20 embryos in **h**) at 2 dpf and levels of *foxj1a* mRNAs were assessed by qPCR. Mean ± SD. ****P < 0.0001 by two-tailed unpaired Student’s *t* test from four biological replicates (three technical replicates each). **j** A cross-section image of the SC of a WT embryo at 2 dpf probed with *fzd7b* riboprobes ventral to the bottom. Arrowhead represents ECs. Scale bar = 15 μm. **k** Embryos were microinjected with control MO, *fzd7b* MO or *fzd7b* MO + *fzd7b* mRNA, and IF stained at 2 dpf with anti-acetylated-α-tubulin antibody. Arrowheads represent motile cilia. Dorsal view anterior to the left. Scale bar = 20 μm. CO: Control. **l** Quantification of the number of cilia per frame in embryos in **k**. Mean ± SD. ****P < 0.0001 by one-way ANOVA with Tukey’s HSD post hoc test (control morphants: *n* = 21 embryos; *fzd7b* morphants: *n* = 21 embryos; *fzd7b* morphants + *fzd7b* mRNA: *n* = 18 embryos; one frame per embryo). **m** *Tg*(*hsp70l:dkk1b-GFP*) embryos at 24 hpf were subjected to heat shock at 39 °C for 1 h, and IF stained at 2 dpf with anti-acetylated-α-tubulin antibody. Arrowheads represent motile cilia. Dorsal view anterior to the left. Scale bar = 20 μm. **n**, **o** Embryos were treated with IWR-1 (10 μM*)* at 8–48 hpf and IF stained with anti-acetylated-α-tubulin antibody only (**n**) or double immunostained with anti-acetylated-α-tubulin antibody and anti-γ-tubulin antibody (**o**) at 2 dpf. Arrowheads represent motile cilia. Dorsal view anterior to the left. The boxed areas in **o** are magnified in the respective lower panels. Scale bar = 20 μm.

We first confirmed the presence of motile cilia in the SC of developing zebrafish by transmission electron microscopy (TEM) and immunofluorescence (IF) staining. TEM revealed the 9 + 2 microtubule configurations, a signature structure of motile cilia, from 2 days post-fertilization (dpf) onward (Fig. 1a and Supplementary Fig. 1). In addition, IF staining of 1-dpf zebrafish embryos with anti-acetylated α-tubulin antibody, which decorates motile cilia, displayed signals in the central SC in which ECs are located (Fig. 1b). To confirm the identity and location of ECs, we carried out IF staining on 2-dpf wild-type (WT) embryos with anti-GFAP antibody (a marker for radial glial cells [RGCs]^12^) and anti-acetylated α-tubulin antibody or on *Tg(foxj1a:egfp*) embryos with anti-GFAP antibody. *foxj1a* is a marker for motile ciliated cells and a master transcription factor of motile ciliogenesis^13,14^. ECs abutted on the ventral central canal (CC) and were distinct from GFAP^+^ RGCs (Supplementary Fig. 2).

Shh, Notch and Wnt signaling pathways are involved in the development of motile cilia^15^. IF staining of *sonic-you* (*syu*^*t4*^) mutant embryos, which harbor a null mutation in the *shh* gene^16^, with anti-acetylated α-tubulin antibody exhibited no noticeable change in cilia staining compared to WT embryos (Fig. 1c, d). Inhibition of Notch signaling in embryos with DAPT, a Notch signaling inhibitor^17^, elicited no obvious change in cilia staining compared to untreated controls (Fig. 1e and Supplementary Fig. 3). These findings suggest that Shh and Notch signaling may not be implicated in the maintenance of ependymal motile cilia. Data available in the literature suggested that of the many *wnt* genes, *wnt4b*, *wnt5b* and *wnt11* (previously called *wnt11r*) may be expressed in ECs^18,19^, which was confirmed by whole-mount in situ hybridization (WISH) with respective riboprobes (data not shown). *wnt4b* single morphants (embryos injected with antisense oligonucleotides morpholino (MO)) and *wnt11* single morphants, but not *wnt5b* single morphants, showed decreased ependymal motile cilia as revealed by IF staining with anti-acetylated α-tubulin antibody. Moreover, *wnt4b/11* double morphants displayed the least number of ependymal motile cilia, which was partially rescued by co-microinjection of *wnt4b/11* mRNAs. However, neither *wnt4b/5b* nor *wnt5b/11* double morphants showed decreased ependymal motile cilia (Fig. 1f, g, Supplementary Figs. 4, 5). WISH on the *wnt4b/11* double morphants with *foxj1a* riboprobes showed increase in the levels of *foxj1a* expression compared to WT embryos (Fig. 1h), which may be a compensatory response. This result was confirmed by quantitative polymerase chain reaction (qPCR) (Fig. 1i). Taken together, these findings indicate that the double morphants have intact ECs with decreased spinal motile cilia. We next examined by WISH the mRNA expression of various *frizzled* (*fzd*) genes that encode receptors for Wnt ligands^20^, and found *fzd7b* expression in the ECs (Fig. 1j and Supplementary Fig. 6). Knockdown of *fzd7b* with MO^21^ diminished ependymal cilia in zebrafish embryos, which was rescued by co-injection of *fzd7b* mRNA (Fig. 1k, l and Supplementary Fig. 7). Furthermore, suppression of Wnt signaling in WT embryos using either *Tg*(*hsp70l:dkk1b-GFP*)^19^ or IWR-1, an Axin stabilizer^22^, diminished ependymal motile cilia (Fig. 1m, n). However, double IF staining of IWR-1 treated embryos with anti-acetylated α-tubulin antibody and anti-γ-tubulin antibody, which stains basal bodies^23^ that are a platform for ciliogenesis^24^, illuminated that IWR-1 did not markedly repress basal bodies in EC (Fig. 1o), suggesting that IWR-1 treatment affects the assembly of motile cilia, but not that of basal bodies. Taken together, these outcomes indicate that Wnt signaling is implicated in the development and/or maintenance of ependymal motile cilia in zebrafish embryos.

### *plcδ3a* is a target gene of Wnt signaling and plays an important role in the maintenance of ependymal motile cilia

**Fig. 2:**
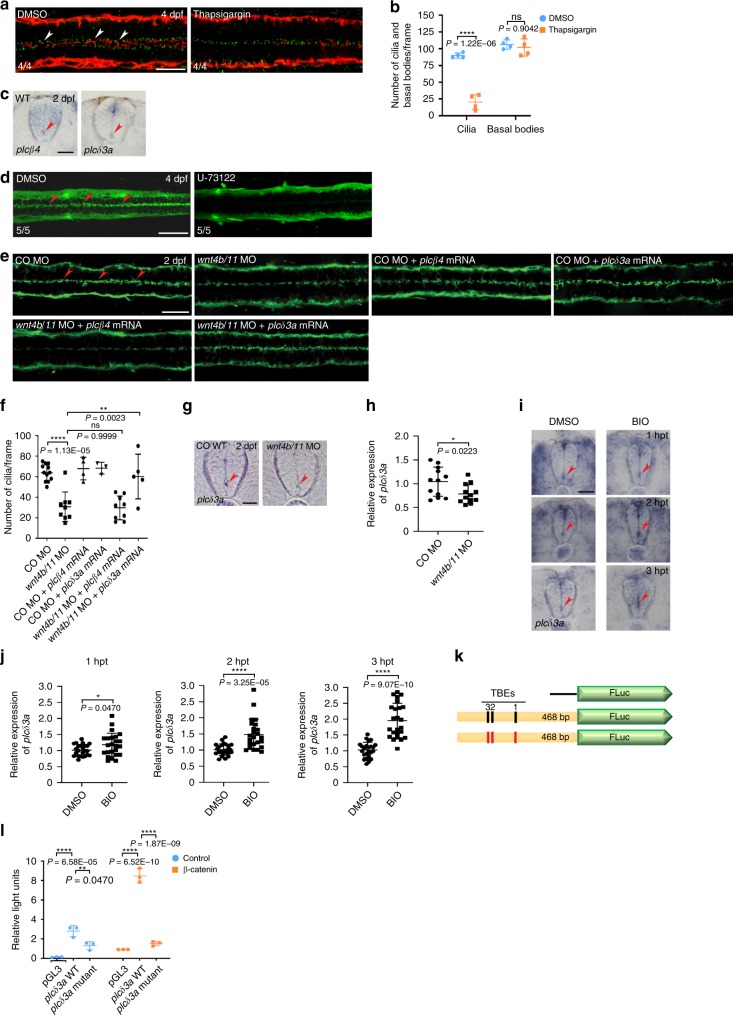
*plcδ3a* is a target gene of Wnt signaling and plays an important role in the maintenance of ependymal motile cilia. **a** DMSO (vehicle control) or thapsigargin (4 nl at 5 μM) was microinjected into the hindbrain ventricles of zebrafish larvae at 4 dpf and the larvae were double immunostained with anti-acetylated α-tubulin antibody (red) and anti-γ-tubulin antibody (green) 4 h after injection. Arrowheads indicate motile cilia. Dorsal view anterior to the left. Scale bar = 20 μm. **b** Quantification of the number of cilia and basal bodies per frame in embryos in **a**. Mean ± SD. ****P < 0.0001 by two-tailed unpaired Student’s *t* test (*n* = 4 embryos per group; one frame per embryo). ns, not significant. **c** Embryos at 2 dpf were probed with *plcβ4* or *plcδ3a* riboprobes and their SCs were cross-sectioned. Images are oriented ventral to the bottom. Arrowheads represent ECs. Scale bar = 20 μm. **d** DMSO (vehicle control) or U-73122 (4 nl at 10 μM) was microinjected into the hindbrain ventricles of zebrafish larvae at 4 dpf and the larvae were IF stained with anti-acetylated α-tubulin antibody 4 h after microinjection. Dorsal view anterior to the left. Scale bar = 20 μm. **e** Control morphants or *wnt4b/11* double morphants were microinjected with *plcβ4* or *plcδ3a* mRNA and immunostained with anti-acetylated α-tubulin antibody at 2 dpf. Arrowheads represent motile cilia. Dorsal view anterior to the left. Scale bar = 20 μm. **f** Quantification of the number of motile cilia per frame in embryos in **e**. Mean ± SD. **P < 0.01 and ****P < 0.0001 by one-way ANOVA with Tukey’s HSD post hoc test (control morphants: *n* = 12 embryos; *wnt4b/11* double morphants: *n* = 9 embryos; control morphants + *plcβ4* mRNA: *n* = 4 embryos; control morphants + *plcδ3a* mRNA: *n* = 3 embryos; *wnt4b/11* double morphants + *plcβ4* mRNA: *n* = 9 embryos; *wnt4b/11* double morphants + *plcδ3a* mRNA: *n* = 5 embryos; one frame per embryo). ns: not significant. **g** Control morphants or *wnt4b/11* double morphants at 2 dpf were probed with *plcδ3a* riboprobes and their SCs were cross-sectioned. Images are oriented ventral to the bottom. Arrowheads point to ECs. Scale bar = 20 μm. **h** RNAs were extracted from each group (20 embryos in **g**) at 2 dpf and levels of *plcδ3a* mRNA were assessed by qPCR. Mean ± SD. *P < 0.05 by two-tailed unpaired Student’s *t* test from four biological replicates (three technical replicates each). CO: Control. **i** Embryos at 45 hpf were treated with DMSO or BIO (5 μM) for 1–3 h and probed with *plcδ3a* riboprobes, and their SCs were sectioned. Images are oriented ventral to the bottom. hpt: hours post-treatment. Arrowhead indicates ECs. Scale bar = 20 μm. **j** Upon BIO treatment, RNAs were extracted from each group (20 embryos in (**i**)) and levels of *plcδ3a* mRNAs were assessed by qPCR. Mean ± SD. *P < 0.05 and ****P < 0.0001 by two-tailed unpaired Student’s *t* test from three biological replicates (eight technical replicates each). **k** Schematic of the *plcδ3a* promoter-firefly luciferase construct used in the luciferase reporter assay. Black rectangles represent three (1–3) Tcf binding elements (TBEs) and red rectangles TBEs with deletions. **l** HEK 293T cells were transfected with WT or mutant *plcδ3a* promoter-firefly luciferase constructs and *Renilla* luciferase plasmid with or without β-catenin plasmid, and processed for dual luciferase assay. Relative Light Units: firefly luciferase activity/*Renilla* luciferase activity. **P < 0.01 and ****P < 0.0001 by one-way ANOVA with Tukey’s HSD post hoc test (*n* = 3 culture replicates per group; each culture was assayed three times).

As Wnt signaling is involved in Ca^2+^ dynamics^25^, we checked whether its alteration by thapsigargin, an inhibitor of the sarco/endoplasmic reticulum Ca^2+^ ATPase^26^, affects ependymal motile cilia. Microinjection of thapsigargin into the hindbrain ventricles of larvae significantly decreased motile cilia compared to larvae microinjected with dimethyl sulfoxide (DMSO), vehicle control. However, significant change in the number of basal bodies was not noted (Fig. 2a, b), demonstrating an important role for Ca^2+^ dynamics in the maintenance of ependymal motile cilia. As Phospholipase C (PLC) is critical to Ca^2+^ dynamics^27^, we investigated the expression of various *plc* genes with WISH and found expression of *plcβ4* and *plcδ3a* in ECs (Fig. 2c and Supplementary Fig. 8). Inhibition of PLC by U-73122, a PLC inhibitor^28^, reduced ependymal cilia (Fig. 2d). In addition, microinjection of *plcδ3a* mRNA into *wnt4b/11* double morphants restored ependymal motile cilia, but microinjection of *plcβ4* RNA did not (Fig. 2e, f). Furthermore, the levels of *plcδ3a* mRNA were diminished in *wnt4b/11* double morphants compared to control morphants as assessed by WISH and qPCR (Fig. 2g, h). We next tested if *plcδ3a* is a target gene of the Wnt signaling by WISH upon treatment with BIO (6-bromoindirubin-3′-oxime), a Wnt signaling activator that inhibits GSK-3^29^. BIO treatment enhanced expression of *plcδ3a* in embryos at 1-3 h post-treatment (hpt) in a time-dependent manner (Fig. 2i, j). In addition, luciferase activity in human embryonic kidney (HEK) 293 T cells transfected with the *plcδ3a* promoter region fused to the *luciferase* gene increased upon co-transfection with β-catenin expression plasmid compared to co-transfection with the respective control plasmid. However, this increase was blunted by a 5-bp deletion of the predicted TCF/LEF binding sites (C/GTTTG/CA/T)^30^ in the promoter region (Fig. 2k, l and Supplementary Fig. 9). Collectively, these findings suggest that *plcδ3a* is a target gene of the Wnt signaling and plays an important role in the maintenance of ependymal motile cilia.

### Cx43 is implicated in the maintenance of ependymal motile cilia

**Fig. 3:**
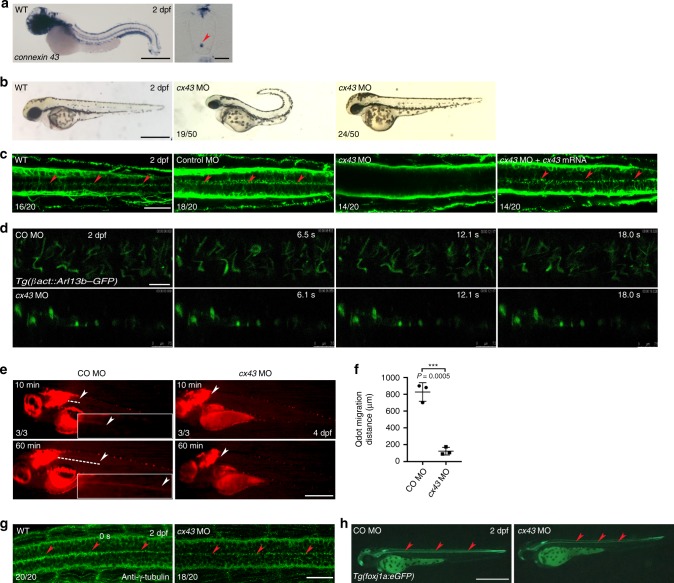
Cx43 is implicated in the maintenance of ependymal motile cilia. **a** WISH of a WT embryo at 2 dpf with *cx43* riboprobes (left). Lateral view anterior to the left. Right panel shows the cross-section image of the SC ventral to the bottom. Arrowhead indicates ECs. Scale bars = left: 650 μm; right: 20 μm. **b** Embryos at one-cell stage were microinjected with *cx43* MO and imaged at 2 dpf. Lateral view anterior to the left. Scale bar = 650 μm. **c** Embryos at one-cell stage were microinjected with either control MO or *cx43* MO or *cx43* MO + mouse *Cx43* mRNA, and IF stained with anti-acetylated α-tubulin antibody at 2 dpf. Arrowheads represent spinal motile cilia. Dorsal view anterior to the left. Scale bar = 20 μm. **d** *Tg*(*bactin2:Arl13b-GFP*) embryos expressing GFP in motile cilia were microinjected at one-cell stage with either control (CO) MO or *cx43* MO and subjected to time-lapse imaging for 2 min (6.7 frames s^−1^) with an intravital multiphoton microscope. Dorsal view anterior to the left. Scale bar = 7.5 μm. **e** Embryos at one-cell stage were microinjected with control MO or *cx43* MO and Qdots were microinjected into the hindbrain ventricles of larvae at 4 dpf, which were then imaged at 10 min and 60 min after the microinjection. Dashed lines indicate migration of Qdots. Arrowheads mark the caudal end of Qdot flow. Lateral view anterior to the left. Scale bar = 650 μm. Insets represent magnifications of the dotted areas. CO: Control. **f** Quantification of Qdot migration distance in the larvae shown in **d**. Mean ± SD. ***P < 0.001 by two-tailed unpaired Student’s *t* test (*n* = 3 larvae per group). **g** Embryos at one-cell stage were microinjected with either control MO or *cx43* MO and immunostained with anti-γ-tubulin antibody at 2 dpf. Arrowheads indicate motile cilia. Dorsal view anterior to the left. Scale bar = 20 μm. **h** *Tg*(*foxj1a:eGFP*) embryos expressing GFP in ECs were microinjected with either control MO or *cx43* MO and imaged at 2 dpf. Arrowheads represent spinal ECs. Dorsal view anterior to the left. Scale bar = 650 μm.

Gap junctions are critical to intercellular Ca^2+^ waves (ICWs)^31–34^. A gap junction is a specialized pore-like intercellular connection that directly links the cytoplasm of neighboring animal cells, thereby coupling them electrochemically, and consists of two connexons (also called hemichannels) on opposing cells. Each connexon is composed of homo- or hetero-hexamers of connexins (Cxs). As previous reports uncovered expression of Cx43 in ECs^35,36^, we probed zebrafish embryos for *cx43* expression. *cx43* was expressed in embryos from one-cell stage onwards and its expression in ECs (ventral SC cells abutting the CC) began from about 42 h post-fertilization (hpf) onwards (Fig. 3a and Supplementary Fig. 10). To check whether Cx43 affects motile cilia, we microinjected *cx43* MO (Supplementary Fig. 11) into embryos and found that at 2 dpf, 38% of embryos showed curved trunks, 48% were morphologically normal (Fig. 3b) and 14% were disintegrated possibly due to injury caused by the microinjection (data not shown). Morphologically normal *cx43* morphants were used in subsequent studies. *cx43* morphants were immunostained with anti-acetylated α-tubulin antibody and displayed reduced cilia, which was recovered by co-microinjection of mouse *Cx43* RNA (Fig. 3c). At stages when *cx43* is not expressed in the SC (24 and 36 hfp), *cx43* MO did not reduce cilia (Supplementary Fig. 12a, b). Furthermore, in *cx43* morphants at 2 dpf, motile cilia were lost in the rostral SC in which *cx43* was expressed, but were relatively spared in the caudal SC where *cx43* was expressed very weakly (Fig. 3a and Supplementary Fig. 12c), suggesting that Cx43 is implicated in the maintenance of cilia rather than ciliogenesis per se.

To visualize the beating of motile cilia in *cx43* morphants, we used *Tg*(*bactin2:Arl13b-GFP*) embryos^37^, in which murine Arl13b fused to green fluorescent protein (GFP) is localized to the ciliary axoneme. Multiphoton imaging of ependymal cilia in live *Tg*(*bactin2:Arl13b-GFP*) embryos microinjected with *cx43* MO revealed decreased beating compared to that in control embryos (Fig. 3d and Supplementary Movies 1, 2). Kupffer’s vesicle (KV) has motile cilia^38^ and *cx43.4* (not related to *cx43*) is expressed in KV^39^. As such, we wished to test if *cx43* is expressed in KV. WISH with *cx43* riboprobes revealed that *cx43* is not expressed in KV (Supplementary Fig. 13).

CSF flow moves dyes injected into the hindbrain ventricle of larvae, which can be used as a functional readout of ependymal motile cilia^40^. To assess the functional consequences of reduced cilia, we microinjected Qdots into the zebrafish hindbrain ventricle and tracked passive migration of Qdots for 60 min. Passive migration of Qdots in *cx43* morphants was practically absent, whereas significant passive migration was observed in control embryos (Fig. 3e, f). Collectively, these findings demonstrate that diminished cilia in *cx43* morphants have decreased function. Meanwhile, *cx43* morphants did not exhibit a decrease in basal bodies, a cilia organizing center found at the base of cilia, as revealed by IF staining with anti-γ-tubulin antibody (Fig. 3g). In addition, microinjection of *cx43* MO into *Tg*(*foxj1a:eGFP*) embryos^13^, in which expression of *eGFP* (*enhanced GFP*) is driven by a promoter of *foxj1a*, did not alter *eGFP* expression in ECs (Fig. 3h). These results indicate that Cx43 affects ependymal motile cilia, but not ECs *per se*. As Foxj1, Rfx2 and Rfx3 transcription factors are implicated in motile ciliogenesis^15^, we wished to check whether Cx43 affects the expression of these three transcription factors in spinal ECs. WISH and qPCR revealed no significant changes in *rfx2* mRNA levels between control and *cx43* morphants, yet uncovered an increase in *foxj1* and *rfx3* levels in *cx43* morphants (Supplementary Fig. 14), which may be a compensatory response. These findings indicate that decreased cilia in *cx43* morphants do not ensue from suppression of the three ciliogenic transcription factors.

**Fig. 4:**
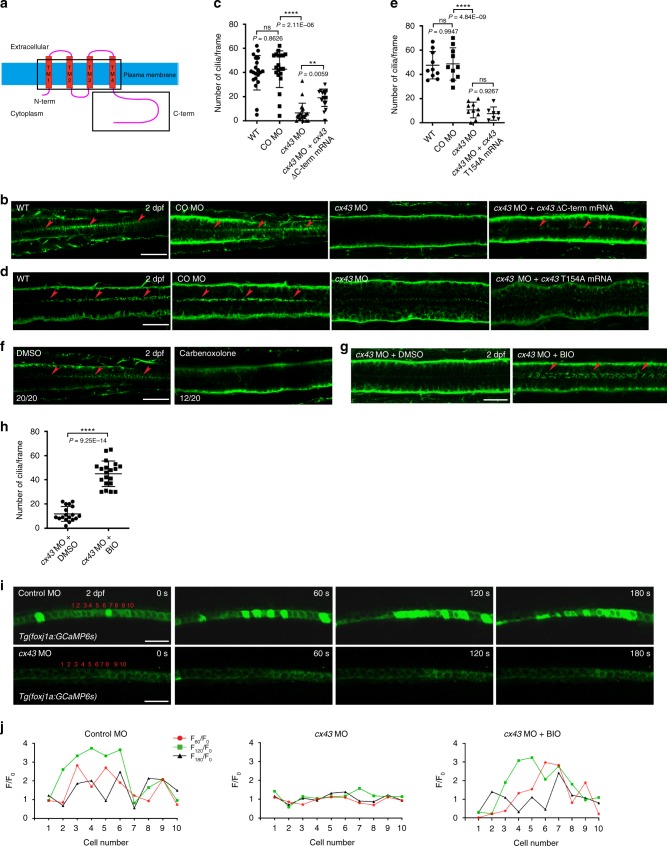
The electrochemical coupling function of Cx43 plays an important role in the maintenance of ependymal motile cilia. **a** Domain structure of Cx43. TM, transmembrane. **b**, **d** Embryos at one-cell stage were microinjected with control MO, *cx43* MO or *cx43* MO + mRNA encoding mouse Cx43 lacking the C-terminal domain (**b**) or *cx43* MO + mRNA that encodes mouse mutant *Cx43* lacking the channel function (**d**), and IF stained with anti-acetylated α-tubulin antibody at 2 dpf. Arrowheads represent motile cilia. Dorsal view anterior to the left. Scale bar = 20 μm. **c**, **e** Quantification of the number of ependymal motile cilia in **b**, **d**, respectively. Mean ± SD. **P < 0.01 and ****P < 0.0001 by one-way ANOVA with Tukey’s HSD post hoc test: **c** *n* = 20 embryos per each group; **e** *n* = 10 embryos per each group except *cx43* MO + *cx43* T154A mRNA (*n* = 7 embryos). ns, not significant. **f** Embryos were treated with carbenoxolone (1 μM) at 18–48 hpf and immunostained with anti-acetylated α-tubulin antibody at 2 dpf. Arrowheads point to motile cilia. Dorsal view anterior to the left. Scale bar = 20 μm. **g** Embryos at one-cell stage were microinjected with *cx43* MO, treated with either DMSO (vehicle control) or BIO (5 μM) at 12–48 hpf, and IF stained with anti-acetylated α-tubulin antibody at 2 dpf. Arrowheads represent motile cilia. Dorsal view anterior to the left. Scale bar = 20 μm. **h** Quantification of the number of ependymal motile cilia per frame in embryos in **g**. Mean ± SD. ****P < 0.0001 by two-tailed unpaired Student’s *t* test (*cx43* MO + DMSO: *n* = 18 embryos; *cx43* MO + BIO: *n* = 20 embryos; one frame per embryo). **i** *Tg(foxj1a:GCaMP6s*) embryos expressing a calcium indicator (GCaMP6s) in ECs were microinjected at one-cell stage with control MO or *cx43* MO, and *cx43* morphants were treated with BIO (5 μM) at 12–48 hpf. Subsequently, they were subjected to time-lapse imaging for 3 min (20 frames/min) with a confocal microscope. Dorsal view anterior to the left. Scale bar = 20 μm. **j** The GFP fluorescence intensity in ten cells (1–10) in each embryo in **i** was individually assessed at 0 (F_0_), 60 (F_60_), 120 (F_120_) and 180 s (F_180_), and presented as F_60_/F_0_, F_120_/F_0_ and F_180_/F_0_.

Cx consists of an N-terminal domain, four transmembrane domains and a C-terminal domain. Both N- and C-termini are located in the cytosol (Fig. 4a), and the four transmembrane domains are responsible for the electrochemical coupling of neighboring cells. The C-terminal domain of Cx possesses regulatory and signaling roles^41,42^. To determine which of the Cx43 domain is associated with the maintenance of motile cilia, we co-microinjected into embryos *cx43* MO and mRNA encoding mouse Cx43 lacking the C-terminal domain^43^ and observed that the ciliary phenotype was rescued (Fig. 4b, c). However, mouse *Cx43 T154A*, a mutant *Cx43* allele that has a missense mutation in the third transmembrane domain resulting in closed gap junctions^44^, did not rescue the ciliary phenotype (Fig. 4d, e). Moreover, treatment of WT embryos with carbenoxolone, a gap junction uncoupler^45^, diminished motile cilia compared to those in DMSO-treated larvae (Fig. 4f). To investigate whether carbenoxolone may be toxic to ECs, we exposed *Tg*(*foxj1a:egfp*) embryos to carbenoxolone and found no discernible decrease in the number of GFP^+^ cells compared to that in control embryos, ruling out the possibility (Supplementary Fig. 15). These findings indicate that the electrochemical coupling function of Cx43, rather than the regulatory and signaling or hemichannel function, plays a more important role in the maintenance of ependymal motile cilia. To test if the ciliary phenotype in *cx43* morphants is associated with changes in Wnt signaling, we treated *cx43* morphants with BIO and noted the rescue of the phenotype (Fig. 4g, h), implying that augmentation of Wnt signaling can maintain motile cilia, at least in part.

As Ca^2+^ is a regulator of motile ciliary beating^46^ and propagates between adjacent cells through gap junctions^32–34^, we wanted to assess Ca^2+^ levels in spinal ECs of *cx43* morphants. To this end, we generated transgenic zebrafish expressing GCaMP6^47^, a Ca^2+^ indicator, driven by *foxj1a* promoter (referred to as *Tg*(*foxj1a:gcamp6s*)). In WT embryos, Ca^2+^ firing in one ependymal cell appeared to propagate to adjacent cells. In *cx43* morphants, however, Ca^2+^ levels in resting ECs were lower than those in WT embryos and the propagation of Ca^2+^ firing was not observed, which was restored to that in WT embryos by BIO treatment (Fig. 4i, j, Supplementary Fig. 16 and Supplementary Movies 3, 4). Collectively, these findings point to a role for Cx43 gap junctions in Ca^2+^ propagation among spinal ECs. ATPs are released from cells through hemichannels (connexons)^48^ that may form in ECs with Cx43, and then could affect Ca^2+^ dynamics in the neighboring cells. As such, we sought to test whether ATP in CSF could regulate motile cilia in ECs. To this end, we microinjected apyrase, which degrades ATP^49^, into the hindbrain ventricles of zebrafish larvae, immunostained them with anti-acetylated α-tubulin antibody, and found no manifest change in ependymal motile cilia (Supplementary Fig. 17). This result implies that Cx43 hemichannels, if formed in ECs, might not be implicated in the maintenance of ependymal motile cilia.

### *cx43* null zebrafish have reduced motile cilia in ECs

**Fig. 5:**
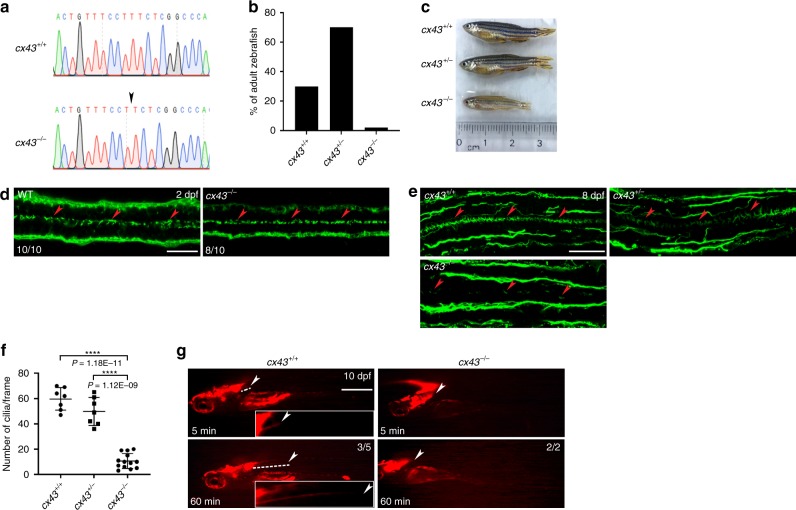
c*x43* null zebrafish have reduced motile cilia in ECs. **a** Electropherograms of the target sequences of the *cx43* gDNA in WT and *cx43*^*−/−*^ zebrafish. Arrow indicates the deletion of the T nucleotide. **b** Zebrafish (*n* = 71) at 2 months post-fertilization (mpf) from mating of *cx43*^*+/−*^ zebrafish were genotyped for *cx43*. **c** Images of 3 mpf zebrafish with the indicated *cx43* genotype. **d** WT or *cx43*^*−/−*^ embryos at 2 dpf were immunostained with anti-acetylated-α-tubulin antibody, imaged with a confocal microscope and genotyped for *cx43*. Arrowheads represent motile cilia. Dorsal view anterior to the left. Scale bar = 20 μm. **e** Larvae at 8 dpf from mating of *cx43*^*+/−*^ zebrafish were cut into cranial and caudal halves. The cranial half was used for *cx43* genotyping, and the caudal half was coronally sectioned at a thickness of 14 μm and then processed for IF staining with anti-acetylated-α-tubulin antibody. Arrowheads represent motile cilia. Dorsal view anterior to the left. Scale bar = 30 μm. **f** Quantification of the number of cilia per frame in embryos in **e**. Mean ± SD. ****P < 0.0001 by one-way ANOVA with Tukey’s HSD post hoc test (*cx43*^*+/+*^ = 7 embryos; *cx43*^*+/−*^ = 7 embryos; *cx43*^*−/−*^ = 13 embryos; one frame per embryo). **g** Qdots were microinjected into the hindbrain ventricles of WT or *cx43*^*−/−*^ zebrafish larvae at 10 dpf and then imaged at 5 and 60 min after the microinjection. Dashed lines indicate passive migration of Qdots. Arrowheads mark the caudal end of Qdot flow. Lateral view anterior to the left. Scale bar = 1 mm. Insets represent magnifications of the dotted areas.

To genetically confirm the ciliary phenotype of *cx43* morphants, we introduced one base pair deletion to the coding sequence of Cx43 using CRISPR/Cas9 technology, yielding a premature stop codon (c.601delT, p.Ser201LeufsTer14; referred to as *cx43*^*−/−*^) (Fig. 5a). Most of *cx43* ^*−/−*^ zebrafish died at 10–14 dpf, especially at 13-14 dpf, and surviving *cx43*^*−/−*^ zebrafish (less than 4%) were sterile and small in body length compared to WT and *cx43*^*+/−*^ siblings (Fig. 5b, c). As we observed a decrease in ependymal motile cilia in *cx43* morphants at 2 dpf, we expected the same decrease in *cx43*^*−/−*^ embryos at 2 dpf. To our surprise, however, we did not see any decrease (Fig. 5d). As zebrafish have many *cx* isoforms, we first suspected that this discrepancy might come from genetic redundancy. To rule out the possibility that the *cx43* MO we used may target other *cx* genes in addition to *cx43*, we examined the 5’ untranslated region (UTR) of all *cx* genes, but we did not find any sequences similar to *cx43* target sequences. We next checked the expression of other *cx* genes in spinal ECs, yet did not observe any other *cx* gene with substantial expression (Supplementary Fig. 18). Finally, this discrepancy could be caused by maternal expression of *cx43*. To avoid maternal effect, we probed *cx43*^*−/−*^ embryos with anti-acetylated α-tubulin antibody at a later stage (8 dpf) and indeed observed decrease in ependymal motile cilia (Fig. 5e, f). Moreover, TEM of 10-dpf zebrafish SCs revealed a decrease in ependymal motile cilia in *cx43*^*−/−*^ embryos with normal morphology and heart beating compared to WT siblings (Supplementary Fig. 19). To assess the functional consequences of reduced cilia, we microinjected Qdots into the hindbrain ventricle of 10-dpf zebrafish and found almost no passive migration of Qdots in *cx43*^*−/−*^ larvae as opposed to significant passive migration in WT siblings (Fig. 5g).

### *Cx43* null mice have reduced motile cilia in ECs

**Fig. 6:**
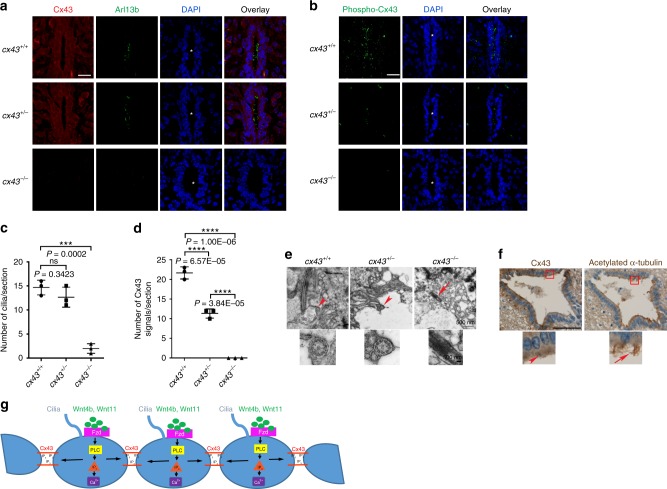
*Cx43* null mice have reduced motile cilia in ECs. **a**, **b** The SCs of P1 pups with the indicated *Cx43* genotype were cross-sectioned, double immunostained with anti-Cx43 and anti-ARL13B antibodies (**a**) or anti-Phospho-Cx43 (Ser386) antibody (**b**), and counterstained with DAPI. Asterisks indicate the central canal (CC). Images are oriented ventral to the bottom. Scale bars = 20 μm. **c**, **d** Quantifications of motile cilia per section in embryos in **a** and Cx43 signals per section in embyos in **b**. Mean ± SD. ***P < 0.001 and ****P < 0.0001 by one-way ANOVA with Tukey’s HSD post hoc test (*n* = 3 mice per group; one frame per mouse). ns, not significant. **e** TEM of the SCs of P1 pups with the indicated *Cx43* genotype. Arrowheads indicate cross-sectioned motile cilia protruding to the CC and arrow marks a cilium-like structure embedded in an EC. Regions marked by arrowheads or arrow are magnified in the respective lower panels. **f** The human spinal cord at C1 level from a cadaver was stained with anti-Cx43 and anti-acetylated-α-tubulin antibodies. The boxed areas are magnified in the respective lower panels. Arrowhead denotes staining of Cx43 and arrow motile cilia. Scale bar = 100 μm. **g** A proposed model for the maintenance of ependymal motile cilia by the Wnt-PLC-IP_3_-Connexin-Ca^2+^ axis.

To investigate whether the ciliary phenotype in *cx43*^*−/−*^ zebrafish recapitulates in *Cx43* knockout (*Cx43*^*−/−*^) mice, we probed the SCs of *Cx43*^*−/−*^ mice pups, which die perinatally^50^, with anti-Cx43 antibody and anti-Arl13b antibody or anti-Phospho-Cx43 (Ser368) antibody, and observed a significant decrease in ependymal motile cilia in *Cx43*^*−/−*^ mice compared to WT and *Cx43*^*+/−*^ siblings. Of note, expansion of the CC was noted in *Cx43*^*−/−*^ mice (Fig. 6a–d). Furthermore, TEM of the SCs in WT and *Cx43*^*+/−*^ mice showed many cross-sectioned motile cilia protruding to the CC, yet no motile cilia protruding to the CC were observed in *Cx43*^*−/−*^ mice. Rarely noted in *Cx43*^*−/−*^ mice were cilia-like structures embedded in ECs (Fig. 6e). Collectively, these findings indicate that Cx43 is required for the maintenance of ependymal motile cilia in mice as well. Finally, immunohistochemistry of the SC from a human cadaver with anti-Cx43 and anti-acetylated α-tubulin antibodies revealed the expression of Cx43 in human ECs (Fig. 6f), demonstrating the conservation of Cx43 expression in ECs from zebrafish to humans.

## Discussion

Here, we show that the Wnt-PLC-IP_3_-Connexin-Ca^2+^ axis is very likely to be required for the maintenance of the ependymal motile cilia in the zebrafish SC (Fig. 6g).

What is the mechanism by which this axis maintains the ependymal motile cilia? Ca^2+^ is the most prevalent cation in vertebrates and is involved in gene expression, muscle contraction, cell motility, apoptosis, synaptic plasticity, and exocytosis^51–53^. Cytosolic Ca^2+^ oscillations have been reported to regulate various biological processes including gene expression^54^. For instance, Lewis and colleagues reported that Ca^2+^ oscillations regulate gene expression mediated by nuclear factor of activated T cells (NFAT), Oct/OAP and NF-kB^55^. Cullen and colleagues demonstrated that the optimized frequency of Ca^2+^ oscillations activates Ras and its downstream extracellular signal-regulated kinase (ERK)/mitogen-activated protein kinase (MAPK) cascade in HeLa cells^56^. Spitzer and colleagues showed that the frequency of Ca^2+^ oscillations can regulate the expression of neurotransmitters in embryonic spinal neurons of *Xenopus laevis*^57^. We showed that the concentration of cytosolic Ca^2+^ in ECs oscillates using *Tg*(*foxj1a:egfp*) embryos and that gap junctions play an important role in the coordination of Ca^2+^ oscillations in adjacent cells and in the generation of Ca^2+^ oscillations. As such, it is tempting to speculate that the propagation of Ca^2+^ oscillations in ECs may be implicated in the expression of genes that are critical to the assembly of motile cilia.

How are Wnt signaling and Cx43 connected to maintain ependymal motile cilia? We come up with two possibilities that are not mutually exclusive. First, Wnt signaling may enhance expression of *cx43*. Fishman and colleagues argued that Wnt1 increases *Cx43* mRNA expression and ICW propagation in neonatal rat ventricular myocytes^58^. We also found that BIO treatment restored number of motile cilia and ICW propagation in *cx43* morphants (Fig. 4g, h, j, Supplementary Fig. 16). Second, Wnt signaling keeps the ECs chemically excitable by maintaining the basal level of ependymal Ca^2+^, which may be required for the ICW propagation through Cx43 gap junctions. Barria and colleagues demonstrated that Wnt signaling controls electrophysiological intrinsic properties of neurons in rat via Ca^2+^ mobilization^59^. As described above, it may be the ICW propagation that induces expression of genes required for motile ciliogenesis. Iovine and colleagues reported that zebrafish homozygous for *cx43* (*short fin* [*sof*]) mutation, but not *cx43*^*+/−*^ zebrafish, showed skeletal phenotypes during zebrafish fin regeneration and that *cx43*^*+/−*^ zebrafish treated with IWR-1 exhibited the skeletal phenotypes^60^, indicating that Cx43 may act upstream of Wnt signaling during the zebrafish fin regeneration. This finding raises the possibility of a positive feedback loop between Wnt signaling and Cx43 in our proposed model (Fig. 6g).

This study focuses on the maintenance of spinal motile cilia at developmental stages after *cx43* is expressed in the SC. Some players in the Wnt-PLC-IP_3_-Connexin-Ca^2+^ axis, especially Wnt signaling and PLC, may be also involved in the initial ciliogenesis. As motile cilia are maintained by balance between cilia assembly (ciliogenesis) and disassembly^61^, it is likely that a possible role for the Wnt signaling and/or PLC in the initial ciliogenesis also contributes to the maintenance of motile cilia after *cx43* is expressed in the SC.

What would be the teleonomic relevance of gap junctions in the maintenance of motile cilia? Motile cilia should beat coordinately to efficiently and unidirectionally move mucus in the respiratory tract and CSF in the brain ventricles and CC, and gap junctions are required for this coordinated beating^15^. Therefore, it is conceivable that beating of motile cilia would become uncoordinated without gap junctions. From a teleonomic perspective, the presence of motile cilia that beat uncoordinatedly does not stand to reason. It does stand to reason, however, that motile cilia are disassembled when coordinated beating is not implemented. Thus, further study is warranted to delineate the mechanism by which the lack of functional gap junctions disassembles motile cilia.

Are all cilia in the CC motile? Two experimental approaches used in this study (IF staining with anti-acetylated α-tubulin antibody and imaging of *Tg*(*bactin2:Arl13b-GFP*) embryos) could not differentiate motile cilia from primary cilia because anti-acetylated α-tubulin antibody labels both primary and motile cilia^62^ and Arl13b exists in both primary and motile cilia^37^. Although analysis of the movie taken from WT *Tg*(*bactin2:Arl13b-GFP*) embryos (Supplementary Movie 1) did not reveal any immotile cilia, this does not necessarily mean that all spinal EC cilia are motile.

CSF-contacting neurons (CSF-cNs; Kolmer–Agduhr (KA) cells in zebrafish) express *foxj1a* and have a kinocilium^63,64^. As such, immunostaining with anti-acetylated α-tubulin antibody cannot tell EC motile cilia from CSF-cNs’ kinocilia. However, the majority of cells surrounding the CC are ECs in zebrafish^63^. Hatler and colleagues argued that a functional leftward fluid flow, which is required for KV to direct L–R asymmetry, entails coordinated ciliary beating and elevated Ca^2+^ within cells. They suggested that communication via gap junctions may coordinate ciliary beating^39^. However, *cx43.4* is not expressed in the zebrafish spinal cord (Supplementary Fig. 18).

Finally, this report underscores the importance of gap junctions in the maintenance of motile cilia and proposes that the augmentation of functional gap junctions by pharmacological or genetic manipulations could mitigate motile ciliopathy.

## Methods

### Reagets

All chemicals were purchased from Sigma-Aldrich, unless indicated otherwise.

### Ethics

Zebrafish experiments were approved by the Chonnam National University Medical School Institutional Animal Care and Use Committee (IACUC; project number: 2017-7). Mouse studies were approved by the Asan Institute for Life Sciences IACUC (project number: 2015-12-158). All animal experiments were conducted in accordance with relevant guidelines and regulations of the Republic of Korea. Written informed consent for the use of tissue from the deceased were obtained from the family of the deceased.

### Zebrafish strains

WT zebrafish (AB strain) were obtained from the Zebrafish International Resource Center, raised in a fish facility under the standard procedures^65^, and staged in dpf or hpf as per standard criteria at 28.5 °C^66^. Zebrafish younger than 1.5 year of age were used in this study and were not selected for gender. The transgenic and mutant lines used in this study were *Tg*(*hsp70l:dkk1b-GFP*)^19^, *Tg*(*βactin2::Arl13b-GFP*)^37^, *Tg*(*foxj1a:eGFP*)^13^ and *sonic-you* (*syu*^*t4*^)^16^.

### DNA manipulation

The plasmid encoding mouse Cx43 (GenBank Accession Number NM_010288.3) was provided by Dr. Myung-Kwan Han and the gene was subcloned into the EcoRI/ClaI sites of the pCS4 + plasmid^67^. To construct a plasmid encoding the mouse Cx43 lacking a C-terminal domain, two DNA fragments (I and II) were PCR-amplified from the mouse Cx43 plasmid with specific primers (Supplementary Table 1) and the two resulting PCR products were cloned simultaneously into the XmaI/Acc65I sites of the mouse Cx43 plasmid. The mouse Cx43 T154A plasmid was generated by site-directed mutagenesis with specific primers (Supplementary Table 1). To generate riboprobes, *cx30.3* (NM_212825), *cx31.7* (XM_001921588), *cx32.2* (NM_001030210), *cx32.3* (NM_199612), *cx34.4* (NM_001130636), *cx34.5* (NM_001030200), *cx35.4* (NM_001017685), *cx40.8* (XM_688906.7), *cx43* (NM_131038), *wnt4b* (NM_131500.1), *wnt11* (NM_131076.2), *plcβ1* (CK146034), *pplcβ4* (XM_002667849.5), *plcδ1a* (NM_001109700.1), *plcδ3a* (NM_001099423.1), *plcδ3b* (XM_005156129.2), *plcδ4a* (XM_688936.5), *plcδ4b* (XM_684872.6), *plch2a* (AW422385.1), *plcl2* (CK706546.1), *plcxd1* (XM_686850), and *plcxd2* (XM_683331.5) were PCR-amplified from zebrafish cDNA with specific primers (Supplementary Table 1) and cloned into pCS2+^68^ or pCS4+^67^ plasmids.

To generate the *plcδ3a* promoter plasmid, the upstream region of the *plcδ3a* coding sequence was PCR-amplified from zebrafish genomic DNA with specific primers (Supplementary Table 2) and cloned into the XhoI/NcoI sites of the pGL3-Basic vector (Promega). Deletion mutants of the *plcδ3a* promoter were generated by site-directed mutagenesis with specific primers (Supplementary Table 2). All plasmids constructed were verified by DNA sequencing analysis (Macrogen).

### Transmission electron microscopy (TEM)

TEM on zebrafish larvae was performed in the Electron Microscopy Facility at the Biomedical Research Institute at Yonsei University College of Medicine as described previously^69^. For TEM of the mice spinal cords (SCs), 16 P1 pups (four WT, nine *Cx43* ^+/−^ and three *Cx43*^*−/−*^) were cryoanesthetized by placing in crushed ice for 5 min and perfused transcardially with 2 ml of heparinized normal saline (0.9% NaCl), followed by 10 ml freshly prepared fixative (2.5% glutaraldehyde, 1% paraformaldehyde (PFA), and 0.1% picric acid in phosphate buffer [PB; 0.1 M, pH 7.4]). Cervical spinal cord (C1 level) was removed and postfixed in the fixative for 2 h at 4 °C. After fixation, tissues were placed in 1% osmium tetroxide in phosphate-buffered saline (PBS [137 mM NaCl, 2.7 mM KCl, 10 mM Na_2_HPO_4_ and 2 mM KH_2_PO_4_]) for 1 h, dehydrated in graded alcohols, embedded in Durcupan ACM (Fluka), and cured for 48 h at 60 °C. Thin sections were cut with a diamond knife and collected on formvar-coated single-slot nickel grids. The grids were counterstained with uranyl acetate and lead citrate and examined on an H-7500 electron microscope (Hitachi) at 80 kV accelerating voltage. Images were acquired using the DigitalMicrograph software driving a SC1000 camera (Gatan) attached to the microscope. Images were saved as TIFF files, and brightness and contrast were adjusted in Photoshop (Adobe Systems).

### Immunohistochemistry of whole-mount zebrafish embryos

Embryos without morphological defect were dechorionated, fixed in 4% PFA for 2 h at room temperature (RT), treated with bleaching solution (3% hydrogen peroxide and 1% KOH in H_2_O) to remove the pigment in the embryos and to permeabilize the tissue, and treated with proteinase K (10 mg ml^−1^; Roche) for 10 min at RT and acetone for 20 min at −20 °C. The embryos were then rinsed in blocking buffer (2.5% bovine serum albumin [BSA] and 2.5% goat serum [Thermo Fisher Scientific] in 1 x PBS), and incubated with anti-acetylated α-tubulin antibody (1:500; catalog number T6793, Sigma), or anti-γ-tubulin antibody (1:200; T6557, Sigma) at 4 °C overnight. After several washes with PBS containing Tween 20 (PBST [PBS in 0.1% Tween 20]), embryos were incubated overnight in the dark at 4 °C with Alexa Fluor 488-conjugated secondary antibody (1:1,000; R37120, Life Technologies).

### Synthesis of antisense riboprobes and capped mRNA

Plasmids encoding the indicated genes were linearized with a restriction endonuclease (New England Biolabs) and then used as templates to synthesize antisense riboprobes using the T7 RNA polymerase (Roche) and a DIG RNA Labeling Kit (Roche) as per the manufacturer’s instructions. Capped mRNAs were synthesized from plasmids linearized with a restriction endonuclease using an mMESSAGE mMACHINE SP6 transcription kit (Thermo Fisher Scientific) according to the manufacturer’s instructions.

### Whole-mount in situ hybridization (WISH)

Embryos without morphological defect were fixed with 4% PFA in PBS at the indicated developmental stages at 4 °C overnight, rinsed with PBST at least three times for 5 min each, transferred to 100% methanol and then stored at −20 °C until further use. The fixed embryos were rehydrated with PBST and treated with proteinase K (10 mg ml^−1^) for 5–30 min depending on the embryo stages. Subsequently, the embryos were postfixed with 4% PFA for 20 min, pre-hybridized with hybridization buffer (50% formamide, 5x saline sodium citrate [SSC] buffer [750 mM NaCl, 75 mM trisodium citrate, pH 7.0; Biosesang], yeast tRNA [500 μg ml^−1^], heparin [50 μg ml^−1^] and 0.1% Tween 20) at 68 °C for 1 h, and then hybridized with DIG-labeled riboprobes at 68 °C overnight with shaking. After washing at 68 °C with 75%, 50%, and 25% formamide in 2x SSC-Tween 20 for 30 min each, 2x SSC-Tween 20 once and 0.2x SSC-Tween 20 four times, the embryos were washed with PBST several times for 5 min each at RT, incubated in 1% blocking buffer (Roche) for at least 1 h on the shaker, and then in anti-digoxigenin antibody (1:2,000; 11277073910, Roche) in 1% blocking buffer overnight. After an extensive wash with PBST, embryos were treated with BM purple AP substrate (Roche) diluted in staining buffer (5 M NaCl, 1 M Tris [pH 9.5], 2 M MgCl_2_ and 0.1% Tween 20) until signal appears. The reaction was stopped by washing with PBST, and the embryos were fixed again with 4% PFA at 4 °C overnight and stored in methanol at −20 °C before imaging.

### Morpholino injections

MOs targeting the start codon of the indicated genes were obtained from Gene Tools and microinjected into one-cell stage zebrafish embryos. The efficiency of MOs targeting the start codon was assessed by the intensity of GFP fluorescence from embryos co-microinjected with MOs and RNAs encoding the MO target N-terminally fused to GFP. To test the efficiency of the MOs, two complementary oligonucleotides harboring the target sequence of the MOs (See below) were annealed and cloned into the BamHI/NcoI sites of the pCS2+-EGFP vector. Upon MO microinjection, embryos with abnormal morphology, if any, were excluded from subsequent experiments.

The following MOs were used:

*wnt4b* MO: 5′-TGGCATCAGATTGCCTGTCTGTC-3′ (translation blocking MO; dose: 4 or 8 ng)

*wnt5b* MO: 5′-GTCCTTGGTTCATTCTCACATCCAT-3′ (translation blocking MO; dose: 4 or 8 ng)

*wnt11* MO: 5′-GGAAGGTTCGCTTCATGCTGTACAA-3′ (translation blocking MO; dose: 4 or 8 ng)

*cx43* MO: 5′-TCCCAACGCACTCCAGTCACCCATC-3′ (translation blocking MO; standard dose: 5 ng)

*fzd7b* MO^21^: 5′-TCGGCTTGTGCTTCGCTGCTATTCC-3′ (translation blocking MO; dose: 5 ng)

*plcδ3a* MO: 5′-GGATTCTTCTTTACCCCAGCATGT-3′ (translation blocking MO; dose: 8 ng)

Standard control MO: 5′-CCTCTTACCTCAGTTACAATTTATA-3′

### Microscopy

For bright-field imaging, a SteREO Discovery V20 stereomicroscope (Zeiss) or a BX50 microscope (Olympus) was used. For confocal imaging, embryos were mounted in 1% low melting temperature agarose (Lonza) on confocal dishes (SPL Life Sciences) and imaged under an LSM 510 Pascal, or an LSM 700 confocal laser scanning microscope (Zeiss). Images were acquired by Zen Black (version 8.1, Zeiss) and assembled using Adobe Photoshop (version CS6). An SP8 intravital multiphoton microscope (Leica) at Korea Basic Science Institute was used to image beating motile cilia in zebrafish embryos, and images were captured using LAS X (Leica)

### Chemical treatment

Carbenoxolone was dissolved in DMSO and administered to embryos at a final concentration of 1.0 μM at 18–48 hpf. BIO (5 μM) and IWR-1 (10 μM), both dissolved in DMSO, were delivered to dechorionated embryos at 12–48 hpf and 8–48 hpf, respectively, in E3 embryo media (5 mM Na Cl, 0.17 mM KCl, 0.33 mM CaCl_2_, 0.33 mM MgSO_4_ and 0.0003% methylene blue). DAPT was dissolved in DMSO, diluted in E3 embryo media to 100 μM, and administered to the dechorionated embryos at 34–48 hpf. Upon chemical treatment, embryos with abnormal morphology, if any, were excluded from subsequent experiments.

### Microinjection into the hindbrain ventricles of zebrafish larvae

Zebrafish larvae at 4 dpf were anesthetized with 1.5 mg ml^−1^ tricaine (3-aminobenzoate methanesulfonate salt) dissolved in blue water (0.0003% methylene blue) and positioned in methyl cellulose (3% in H_2_O). Qdot (4 nl, Thermo Fisher Scientific), thapsigargin (4 nl of 5 μM), apyrase (0.175 U) or U-73122 (4 nl of 40 μM) was microinjected into the hindbrain ventricles of zebrafish larvae. The microinjection was implemented at 4 dpf due to difficulty in ventricle microinjection at 2 dpf.

### Quantitative PCR (qPCR)

Total RNAs were extracted from 2-dpf embryos using TRIzol (Molecular Research Center). cDNAs were synthesized from the extracted RNAs using SuperiorScript III Master Mix (Enzynomics) as per the manufacturer’s instructions and then mixed with the indicated primers and TOPreal qPCR 2x PreMIX (Enzynomics). Subsequently, qPCR was performed on a Rotor-Gene Q Real-Time PCR cycler (Corbett Research). Primers for *18* *S rRNA*, *actb1* or *gapdh* were used for normalization. The sequences of the primers used are listed in Supplementary Table 3.

### Luciferase reporter assay

HEK 293T cells were maintained in RPMI1640 media (Welgene) supplemented with 10% fetal bovine serum (FBS, Welgene) and 1x Antibiotic-Antimycotic (Thermo Fisher Scientific). The cells were maintained at 37 °C and 5% CO_2_ atmosphere and regularly passaged once they reached >80% confluency. HEK 293 T cells were seeded onto 24-well plates at a density of 5 × 10^4^ cells/well. When the cells reached about 60% confluency, they were transfected with the pGL3-Basic plasmids (50 ng), pCS2+ plasmid (50 ng), WT or mutant reporter plasmids (50 ng), or plasmids encoding β-catenin^70^ (25 ng) using Lipofectamine 3000 (Thermo Fisher Scientific) according to the manufacturer’s protocol. The pEGFP-C1 plasmid (Clontech) was used to monitor the transfection efficiency. At 24 h post-transfection, the media was removed, and the cells were rinsed with 500 µl of Dulbecco’s PBS (DPBS) thoroughly, treated with 100 µl of 1× Passive Lysis Buffer (Promega) and rocked for 15–20 min. The dual luciferase assay was carried out using a Dual-Luciferase Reporter Assay System (Promega) and an Orion L Microplate Luminometer (Titertek-Berthold). The cell lysate (20 µl) was mixed with the Luciferase Assay Buffer II plus Luciferase Assay Substrate (100 µl), and the firefly luciferase activity was measured for 10 s after a 3-sec delay. Subsequently, Stop & Glo Buffer plus Stop & Glo Buffer Substrate (100 µl) was added and the Renilla luciferase activity was measured for 10 s after a 3-s delay. The ratio of firefly luciferase activity to *Renilla* luciferase activity was expressed as relative light unit.

### Serial sectioning of zebrafish embryos and larvae

Embryos and larvae were fixed with AB fix solution (8% PFA and 2× fix solution [8% sucrose and 0.03% 1 M CaCl_2_ in PBS]) overnight, embedded in 1.5% agarose dissolved in 5% sucrose, and kept in a sealed 15-ml tube with 30% sucrose in PBS overnight. Subsequently, agarose blocks were frozen in butanol (Junsei Chemical) chilled with liquid nitrogen and then serially sectioned into slices using a CM3000 S microtome (Leica).

### Immunohistochemistry of sectioned zebrafish tissues

Slides with sectioned tissues were dried for at least 2 h at RT and then soaked in PBS for 1 h in a glass jar. The slides were mounted on a tray, washed three times with PBS for 5 min each, blocked with blocking buffer (2.5% BSA and 2.5% goat serum in PBS) for 1–2 h, and treated with anti-acetylated-α-tubulin antibody (1:500), or anti-GFAP antibody (1:200, GTX128741, GeneTex) at 4 °C overnight. The sections were then washed 10 times with PBS for 5 min each, blocked for 1 h, and then incubated in the dark with Alexa Fluor 488-conjugated secondary antibody (1:1,000) at 4 °C overnight. After five washes with PBS, the sections were incubated with 4′,6-diamidino-2-phenylindole (DAPI, 10.9 μM) for 1 min at RT and washed with PBS three times for 5 min each.

### Generation of *Tg(foxj1a:GCaMP6s)* zebrafish

*Tg*(*foxj1a:GCaMP6s*) construct was generated using a Gateway cloning kit (Thermo Fisher Scientific) as per the manufacturer’s instructions. GCaMP6s was PCR-amplified from the pGP-CMV-GCaMP6s plasmid^71^ with specific primers harboring attB sequences (fwd: 5′-GGG GAC AAG TTT GTA CAA AAA AGC AGG CTC CAT GGG TTC TCA TCA TCA TC-3′; rev: 5′-GGG GAC CAC TTT GTA CAA GAA AGC TGG GTG TCA CTT CGC TGT CAT CAT TTG-3′). The resulting PCR product was recombined with the donor vector, pDONR 211 (Thermo Fisher Scientific), using BP Clonase II Enzyme Mix (Thermo Fisher Scientific). The resulting entry clone, 5′ element plasmid (the *foxj1a* promoter plasmid provided by Dr. Brian Ciruna)^13^ and p3E-polyA were simultaneously transferred to the destination vector pDestTol2pA2^72^ using LR Clonase II Enzyme Mix (Thermo Fisher Scientific). The final construct, *Tg*(*foxj1a:GCaMP6s*), was microinjected into one-cell stage embryos, which were raised to adulthood.

### Real-time imaging of Ca^2+^ and motile cilia in spinal ECs of zebrafish

*Tg(foxj1a:GCaMP6s*) embryos at 2 dpf were mounted onto confocal dishes in 1% low-melting temperature agarose, and time series images were taken with an LSM 700 confocal microscope for 3 min (20 frames/min). The Ca^2+^ change in each EC was measured by ImageJ (National Institutes of Health). *Tg*(*bactin2:Arl13b-GFP*)^37^ embryos at 2 dpf were mounted onto confocal dishes in 1% low-melting temperature agarose and then imaged under an intravital multiphoton microscope for 2 min (6.7 frames s^−1^).

### Generation of *cx43*^*−/−*^ zebrafish

*cx43*^*−/−*^ were generated via CRISPR/Cas9 technology^73,74^. The *cx43* guide RNA targeting the second exon was designed with ZiFiT Targeter (http://zifit.partners.org*)*. To generate the *cx43* guide RNA, oligonucleotides were annealed (fwd: 5′-TAG GGT GGA CTG TTT CCT TTC T-3′; rev: 5′-AAA CAG AAA GGA AAC AGT CCA C-3′), cloned into the pT7-gRNA vector^75^, and in vitro transcribed with an mMESSAGE mMACHINE T7 transcription kit (Thermo Fisher Scientific). The plasmid encoding Cas9 optimized for zebrafish codon^76^ was digested with XbaI, and *cas9* RNA was synthesized using an mMESSAGE mMACHINE T3 transcription kit (Thermo Fisher Scientific). Finally, the *cx43* guide RNA (12.5 ng) and the *cas9* RNA (300 ng) were co-microinjected into one-cell stage zebrafish embryos.

### Genotyping

For zebrafish genotyping, genomic DNA (gDNA) was isolated as described previously^77^. In brief, tail fins of adult zebrafish were clipped upon anesthetization with tricaine. The clipped fin or embryos were incubated with 50 mM NaOH (50–200 μl), heated to 95 °C until tissues were dissolved, and cooled down to 4 °C. Subsequently, 1/10 volume of 1 M Tris-HCl (pH 8.0) was added to neutralize the basic solution. After centrifugation at 16,363 × *g* for 10 min, the resulting supernatant was used for PCR. The primer pair used to PCR-amplify zebrafish *cx43* was 5′-CGC ACC TAC ATC TTC AGC ATC AT-3′ (forward) and 5′-ACG CGG TCC TTG ATT CGT TTG A-3′ (reverse). For mice genotyping, gDNA was extracted from mice ear punches using a Wizard Genomic DNA Purification Kit as per the manufacturer’s instructions (Promega). The primer pair to detect WT mice *Cx43* was 5′-AGG TGG TGT CCA GAG CCT TA -3′ (forward) and 5′- CAC GTG AGC CAA GTA CAG GA-3′ (reverse), and the resulting PCR product size was 600 bp. The primer pair to detect mutant mice *Cx43* was 5′- AGG TGG TGT CCA GAG CCT TA -3′ (forward) and 5′- AAT CCA TCT TGT TCA ATG GCC GAT C -3′ (reverse), and the resulting PCR product size was 320 bp.

### Immunohistochemistry of the zebrafish SCs

Zebrafish larvae were anesthetized by 200 μg ml^−1^ of tricaine until movement ceased and were then fixed in 4% PFA overnight at 4 °C. Fixed embryos were embedded in 1.5% agar blocks containing 5% sucrose and equilibrated in 30% sucrose solution. Frozen blocks were sliced into horizontal sections at a thickness of 14-µm using a cryostat microtome (Microm HM 525, Thermo Fisher Scientific) and mounted on glass slides. The resulting sections were rinsed with PBS several times and then blocked in 5% BSA (Biosesang) with sheep serum (Jackson ImmnoResearch). Subsequently, the sections were treated with anti-acetylated α-tubulin (1:500) antibody overnight at 4 °C, washed for 1 h with PBS and treated with Alexa Fluor 488-conjugated secondary antibodies (1:1000) overnight at 4 °C followed by treatment with DAPI (1:1,000) for 15 min and washing for 1 h with PBS. Images were obtained from the sections of *cx43*^*−/−*^ larvae and their siblings.

### Immunohistochemistry of the mice spinal cords (SCs)

The SCs were separated from P1 WT, *Cx43*^+/−^ and *Cx43*^*−/−*^ mice, embedded into paraffin after rinse in 30% sucrose in PBS, and sectioned at a thickness of 4 μm. The resulting sections were incubated at RT with mouse anti-CX43 antibody (1:10; 13-8300, Life Technologies), rabbit anti-Phospho-Cx 43 (Ser368) antibody (1:100; 3511, Cell Signaling Technology) or rabbit anti-ARL13B antibody^78^ (1:800, a gift from Dr. Hyuk Wan Ko) overnight, overnight or for two hr, respectively, washed three times with PBS for 30 min each and blocked with the blocking buffer for two hr. Subsequently, the sections were incubated with mouse Alexa Fluor 488-conjugated (1:1000) or rabbit Alexa Fluor 555-conjugated secondary antibodies (1:1000; A32732, Life Technologies), respectively, for 1 h at RT in the dark. Nuclei were counterstained with DAPI (10.9 μM) for 1 min at RT. Subsequently, the sections were washed three times in TBST (50 mM Tris, 150 mM NaCl and 0.1% Tween 20), mounted in Antifade Mounting Medium (Vector Laboratories) and imaged under a microscope.

### Immunohistochemistry of the human SC

The SC from a cadaver (22-year-old Korean male) was embedded in paraffin, deparaffinized and pre-treated with citrate buffer (10 mM, pH 6.0) to retrieve antigen. After blocking the endogenous peroxidase activity with 3% hydrogen peroxide (H_2_O_2_ and 3% horse serum albumin [Thermo Fisher Scientific]), tissue sections were consecutively incubated with anti-Cx43 antibody (1:500) or anti-acetylated α-tubulin antibody (1:1,000) overnight at 48 °C, biotinylated secondary antibodies (Agilent Technologies), streptavidin-conjugated horseradish peroxidase (Vector Laboratories) and 3,3′-diaminobenzidine (DAB) until the desired stain intensity appeared. The sections were finally counterstained with hematoxylin.

### Statistics and reproducibility

Data are presented as mean ± standard deviation (SD). Error bars indicate SD. The *P* value was determined by either the one-way ANOVA with Tukey’s honest significant difference (HSD) post hoc test or the two-tailed unpaired Student’s *t* test. Microsoft Excel (version 2013) and GraphPad Prism (version 5.01) were used for statistical analysis. All experiments in this study were repeated independently at least twice with similar results.

### Reporting summary

Further information on research design is available in the Nature Research Reporting Summary linked to this article.

## Supplementary information

Supplementary Information

Description of Additional Supplementary Files

Supplementary Movie 1

Supplementary Movie 2

Supplementary Movie 3

Supplementary Movie 4

Reporting Summary

## Data Availability

The authors declare that the data supporting the findings of this study are available within the paper and its supplementary information files. Reagents are available upon request. The source data underlying all figures in which statistics are represented are provided as a Source Data file.

## Source data

Source Data
