# Supplementary material for: Wnt-PLC-IP3-Connexin-Ca2+ axis maintains ependymal motile cilia in zebrafish spinal cord
Source: Nat Commun. 2020 Apr 20;11:1860. doi: 10.1038/s41467-020-15248-2 (PMC7170879; doi:10.1038/s41467-020-15248-2)
Supplement: Supplementary file 1 — Supplementary Information [file 41467_2020_15248_MOESM1_ESM.pdf]

Supplementary Information

Wnt-PLC-IP_3-Connexin-Ca^{2+} axis maintains ependymal motile cilia in zebrafish spinal cord

Zhang et al.

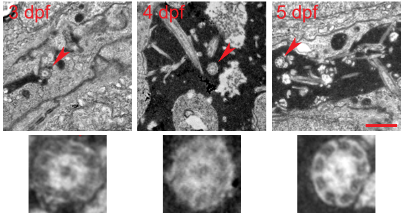

Supplementary Figure 1. TEM of the SCs of zebrafish embryos at 3-5 dpf. Arrowheads indicate motile cilia with the 9 + 2 microtubule configurations, which are magnified below. Scale bar = 1 μm.

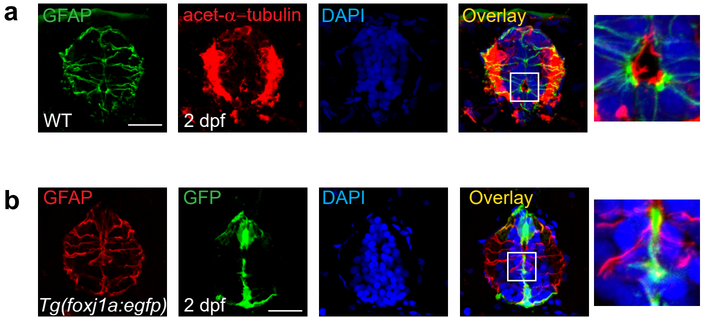

Supplementary Figure 2. Ependymal cells are distinct from GFAP+ radial glial cells. (**a**) WT embryos at 2 dpf were cross-sectioned, double-immunostained with anti-GFAP and anti-acetylated-α-tubulin antibodies and counterstained with DAPI. (**b**) *Tg(foxj1a:egfp)* embryos at 2 dpf were cross-sectioned, immunostained with anti-GFAP antibody and counterstained with DAPI. Images are oriented ventral to the bottom. The boxed areas are magnified to the right. Scale bars = 20 μm.

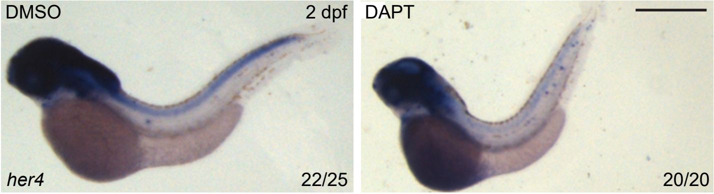

Supplementary Figure 3. DAPT treatment suppresses expression of *her4*. Embryos were treated with DMSO or DAPT (100 μM) for 34-48 hpf and probed with riboprobes of *her4,* a target gene of Notch signaling. Lateral view anterior to the left. Scale bar = 450 μm.

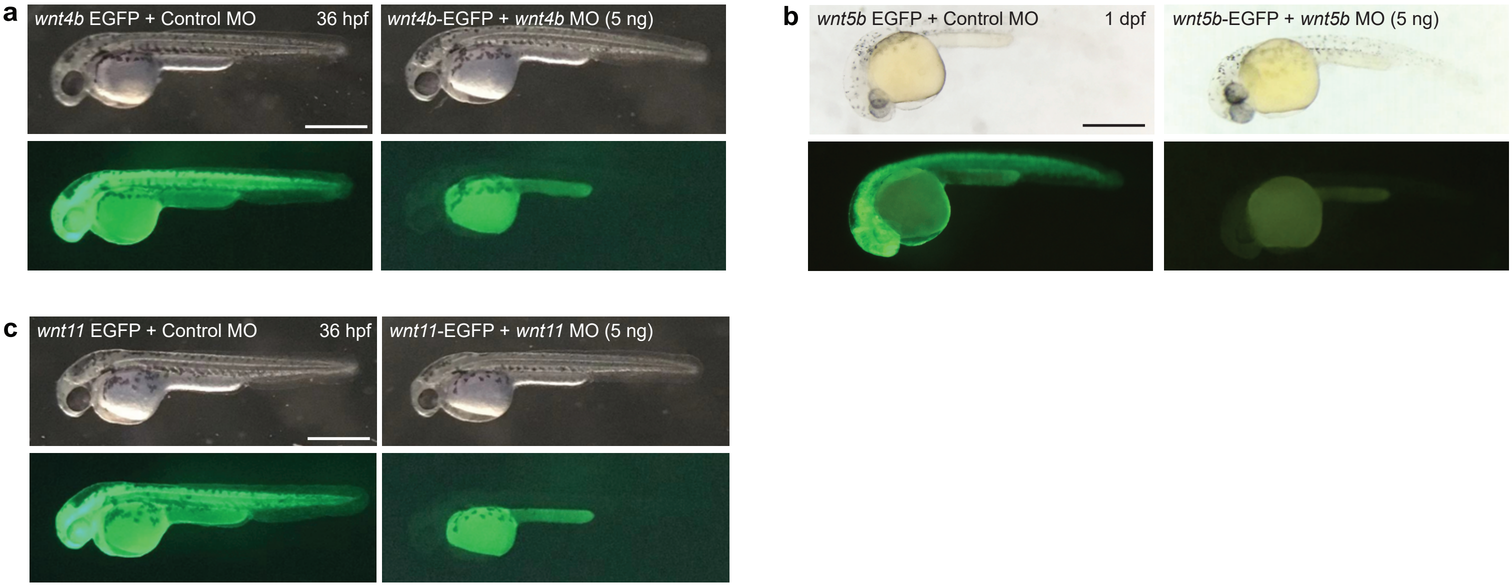

Supplementary Figure 4. Efficiency tests of MOs targeting *wnt4b*, *wnt5b* or *wnt11*. (**a-c**) One-cell stage embryos were co-microinjected with indicated MO (5 ng) + RNA encoding EGFP fused to target sequences of indicated *wnt*, and imaged at 1 dpf (**b**) or 36 hpf (**a,c**). Upper panels are bright field images and lower panels fluorescent images. Scale bar = 400 μm.

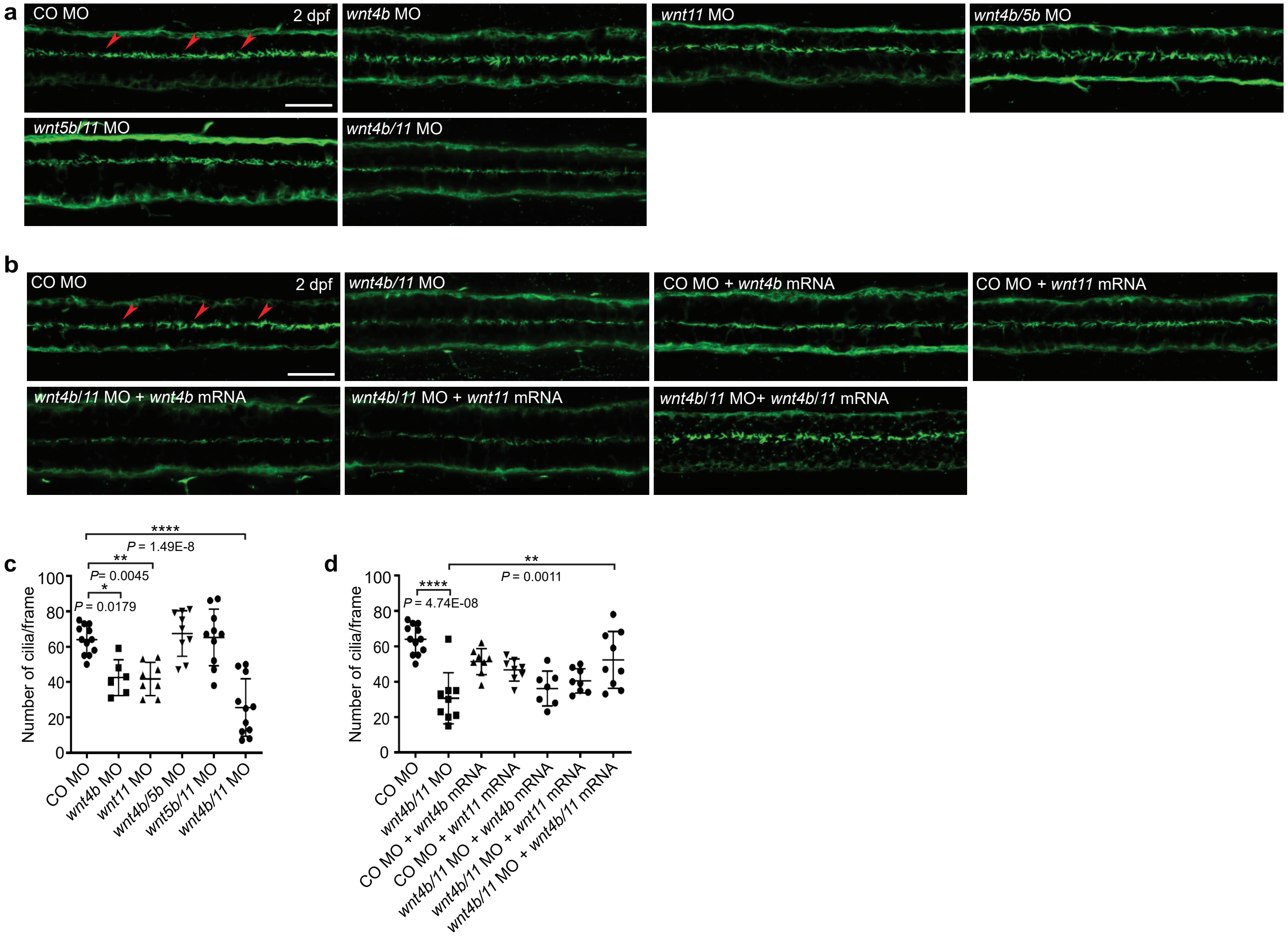

Supplementary Figure 5. *wnt4b/11* double morphants decrease the number of ependymal motile cilia. (**a, b**) Embryos were microinjected with indicated MO(s) alone (**a**) or along with indicated mRNA(s) (**b**), and immunostained at 2 dpf with anti-acetylated-α-tubulin antibody. MO dose: single MO = 8 ng; double MOs = 4 ng each. mRNA dose: single mRNA = 30 ng; double mRNA = 20 ng each. Arrowheads represent motile cilia. Dorsal view anterior to the left. Scale bar = 20 μm. (**c, d**) Quantification of the number of cilia per frame in embryos in (**a, b**), respectively. Mean ± SD. * $P < 0.05$, ** $P < 0.01$ and **** $P < 0.0001$ by one-way ANOVA with Tukey's HSD post hoc test. (**c**) n: CO MO = 12; *wnt4b* MO = 6; *wnt11* MO = 8; *wnt4b/5b* MO = 9; *wnt5b/11* MO = 10; *wnt4b/11* MO = 11. One frame per embryo. (**d**) n: CO MO = 12; *wnt4b/11* MO = 9; CO MO + *wnt4b* mRNA = 8; CO MO + *wnt11* mRNA = 8; *wnt4b/11* MO + *wnt4b* mRNA = 7; *wnt4b/11* MO + *wnt11* mRNA = 8; *wnt4b/11* MO + *wnt4b/11* mRNA = 8. One frame per embryo.

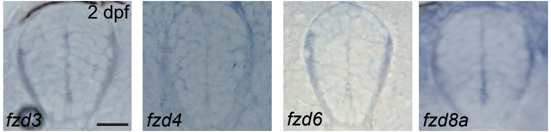

Supplementary Figure 6. Cross-section images of the SC of embryos at 2 dpf probed with the indicated *fzd* riboprobes. Ventral to the bottom. Scale bar = 15 µm.

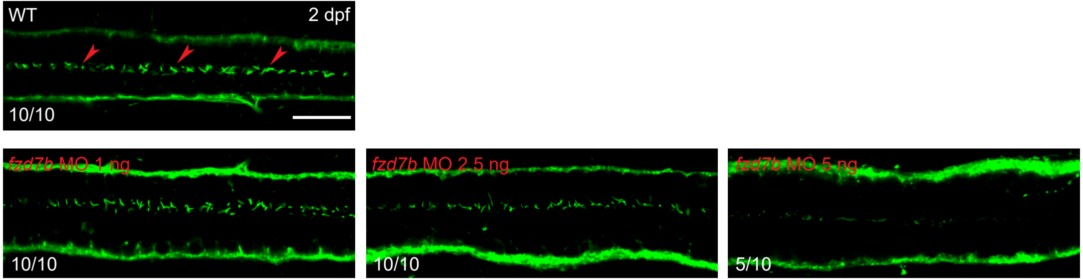

Supplementary Figure 7. Effect assessment of *fzd7b* MO. One-cell stage embryos were microinjected with the indicated dose of *fzd7b* MO and immunostained at 2 dpf with anti-acetylated-α-tubulin antibody. Arrowheads represent spinal motile cilia. Dorsal view anterior to the left. Scale bar = 20 μm.

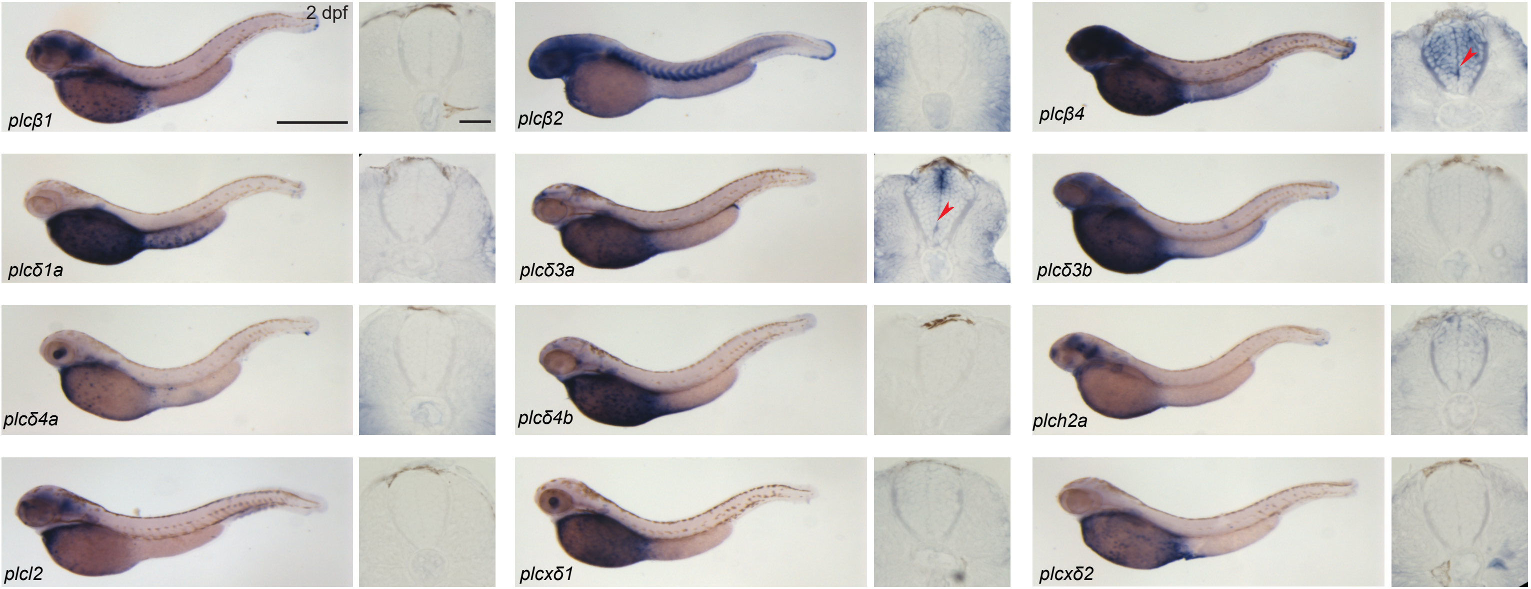

Supplementary Figure 8. Expression of *plcβ4* and *plcδ3a* in ECs. Embryos at 2 dpf were probed with the indicated *plc* riboprobes. Lateral view anterior to the left. Cross-sectioned images presented ventral to the bottom are shown to the right. Arrowheads represent ECs. Scale bars = left: 650 μm; right: 20 μm.

```
-468                             GAACACAA  GACCAAAAAA  TATGTTATAG  AATATGGAAT  ATTCACTATC
-420 ATTATGTCTC  TAACATTCTA  ACATTAATGT  CTAACATTCT  TTAATATTTC  TACCTTTTTG  TTCAAGATAA
-350 TGAAAGAAAC  TCAAGCACCT  TGGAATTAAG  TGGAGAATGA  GTAAATAATG  ACAAAATTTG  CATCCACATA
      TBEs 2 & 3
-280 TAAGAATACT  TACTAACGTA  ATACGTATAT  ATATATAATT  ATTATTATTA  TTATTCCATA  GGAATATATG
-210 TCTGATGAGA  TTGAAGGAGT  GAACTAAAAT  TCTCATTACC  ACGCGCATGT  CCGTGAAACT  TGAATATTCA
                                                                  TBE 1
-140 TATCCGCCCA  CTGACACCGA  AAATCTCAGA  AACCAAAAAA  CTCGTAACAC  CTTCATGAAT  AAACCCAACA
-70  GGCGGAGTCC  AAACAAGTCA  CAAGCCTGTG  ACAAGTTCAT  GTCCTGCATT  TAAACTTTTT  TATCGCAACA

        TSS
 +1
```

Supplementary Figure 9. Promoter sequences of *plcδ3a* with three TBEs (Tcf binding elements). In red are deleted nucleotides in the TBEs of the mutant *plcδ3a* promoter. TSS, transcriptional start site.

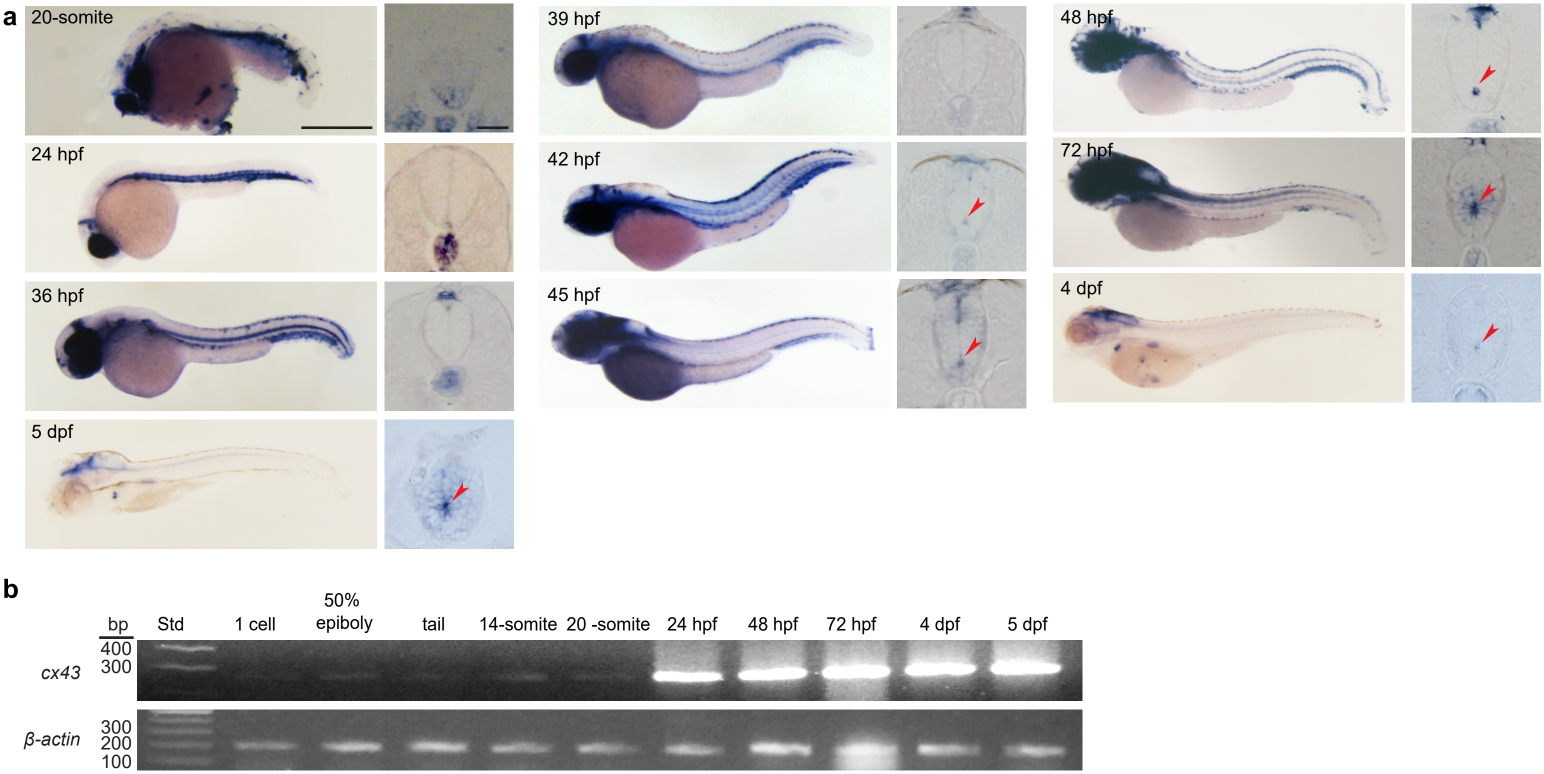

Supplementary Figure 10. Expression analysis of *cx43* in zebrafish embryos and larvae. (a) WISH with *cx43* riboprobes of zebrafish from 20-somite stage to 5 dpf. Lateral view anterior to the left. Right panels show the cross-sectioned images of the SCs ventral to the bottom. Arrowheads indicate ECs. Scale bars = left: 650 μm; right: 20 μm. (**b**) RNAs were extracted from zebrafish at the indicated stages, converted to cDNA, and amplified by PCR with *cx43* primers. Cropped gel pictures are presented. ß-actin was used as loading control. bp, base pairs; Std, DNA standards.

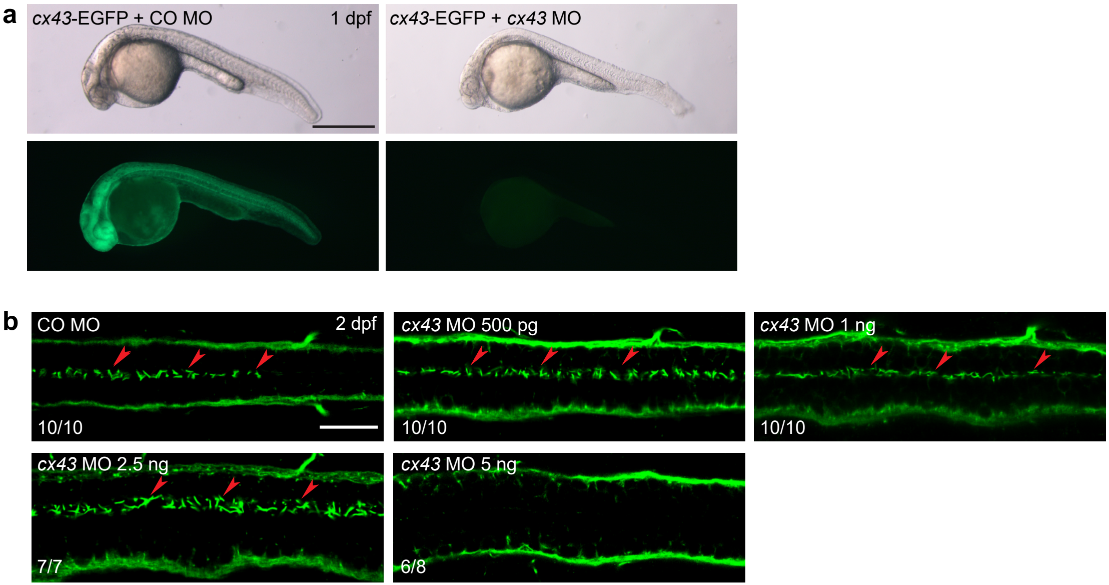

Supplementary Figure 11. Effect assessment of *cx43* MO. (**a**) One-cell stage embryos were co-microinjected with control (CO) MO or *cx43* MO (5 ng) plus RNA encoding EGFP fused to target sequences of *cx43* MO, and imaged at 1 dpf. Upper panels are bright field images and lower panels fluorescent images. Scale bar = 400 μm. (**b**) Embryos at one-cell stage were micro-injected with control MO or indicated dose of *cx43* MO, and immunostained with anti-acetylated α-tubulin antibody at 2 dpf. Arrowheads represent motile cilia. Dorsal view anterior to the left. Scale bar = 20 μm.

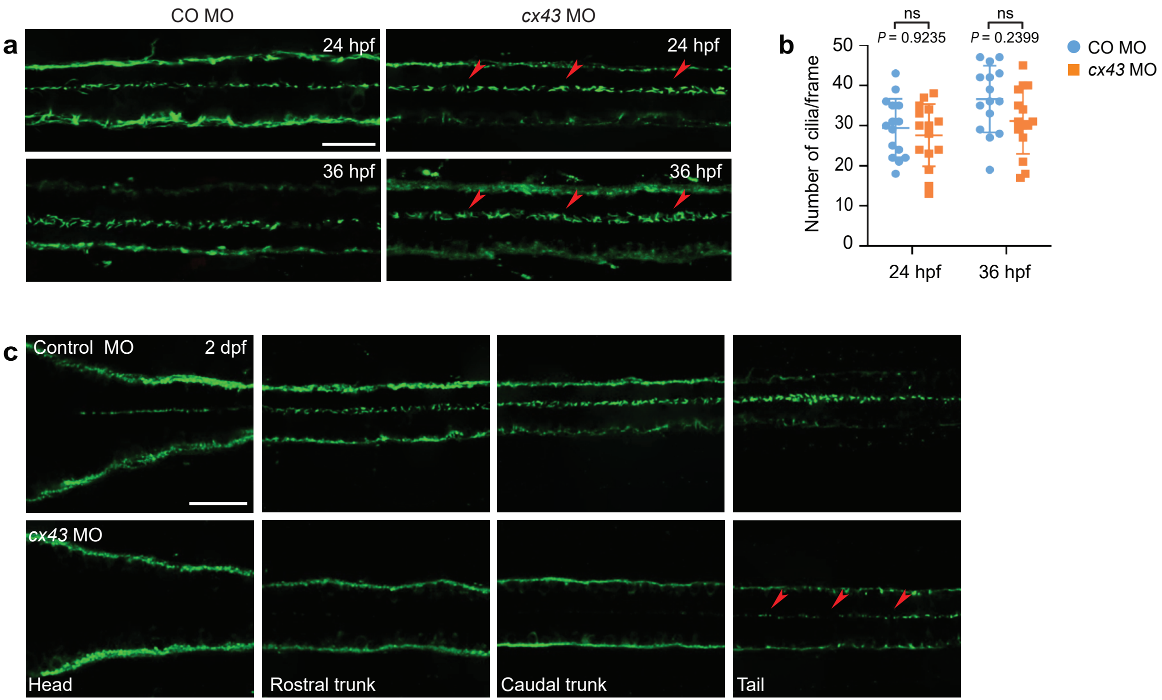

Supplementary Figure 12. Effect of *cx43* MO on ependymal motile cilia depends on *cx43* expression. (**a**) Embryos at one-cell stage were microinjected with control (CO) MO or *cx43* MO, and immunostained with anti-acetylated α-tubulin antibody at 24 or 36 hpf. Arrowheads represent motile cilia. Dorsal view anterior to the left. Scale bar = 20 μm. (**b**) Quantification of the number of cilia per frame in embryos in (**a**). Data are presented as Mean ± SD. ns (not significant): $P > 0.05$ by two-tailed unpaired Student's *t*-test (n = 15 embryos per group; one frame per embryo). hpf, hours post-fertilization. (**c**) Embryos at one-cell stage were microinjected with either control MO or *cx43* MO, and IF stained at 2 dpf with anti-acetylated-α-tubulin antibody. Arrowheads represent motile cilia. Dorsal view anterior to the left. Scale bar = 20 μm.

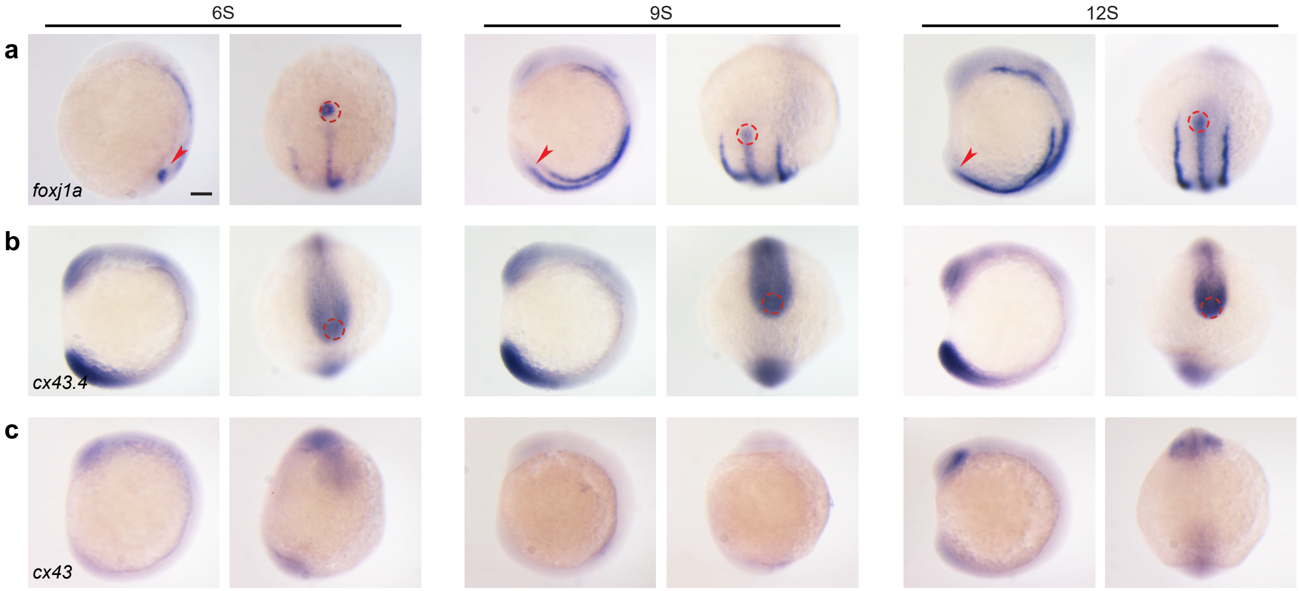

Supplementary Figure 13. ***cx43*** **is not expressed in Kupffer's vesicle (KV).** (**a-c**) WISH with indicated riboprobes of zebrafish embryos at 6-somite (6S), 9S and 12S stages. *foxj1a* (**a**) and *cx43.4* (**b**) riboprobes are markers of KV. Left panels are lateral view anterior to the top. Right panels are dorsal view anterior to the top. Arrowheads and circles indicate KVs. Scale bar = 100 μm.

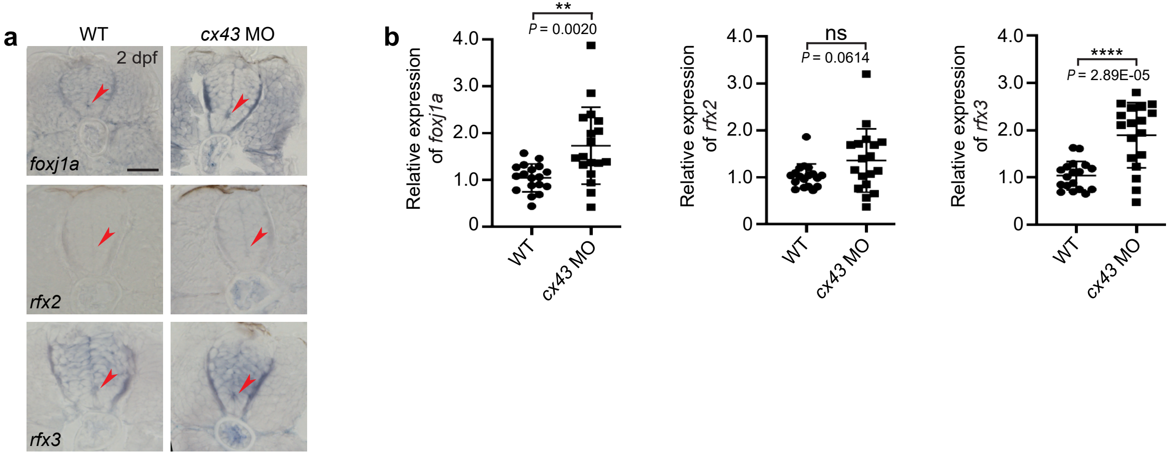

Supplementary Figure 14. An increase in *foxj1* and *rfx3* levels in *cx43* morphants. (**a**) Embryos at one-cell stage were microinjected with *cx43* MO, probed at 2 dpf with the indicated riboprobes, and cross-sectioned. Images are oriented ventral to the bottom. Arrowheads indicate ECs. Scale bar = 20 μm. (**b**) RNAs were extracted from each group (20 embryos in (**a**)) at 2 dpf and levels of indicated mRNAs were assessed by qPCR. Mean ± SD. ** $P < 0.01$ and **** $P < 0.0001$ by two-tailed unpaired Student's *t*-test from three biological replicates (six technical replicates each). ns, not significant.

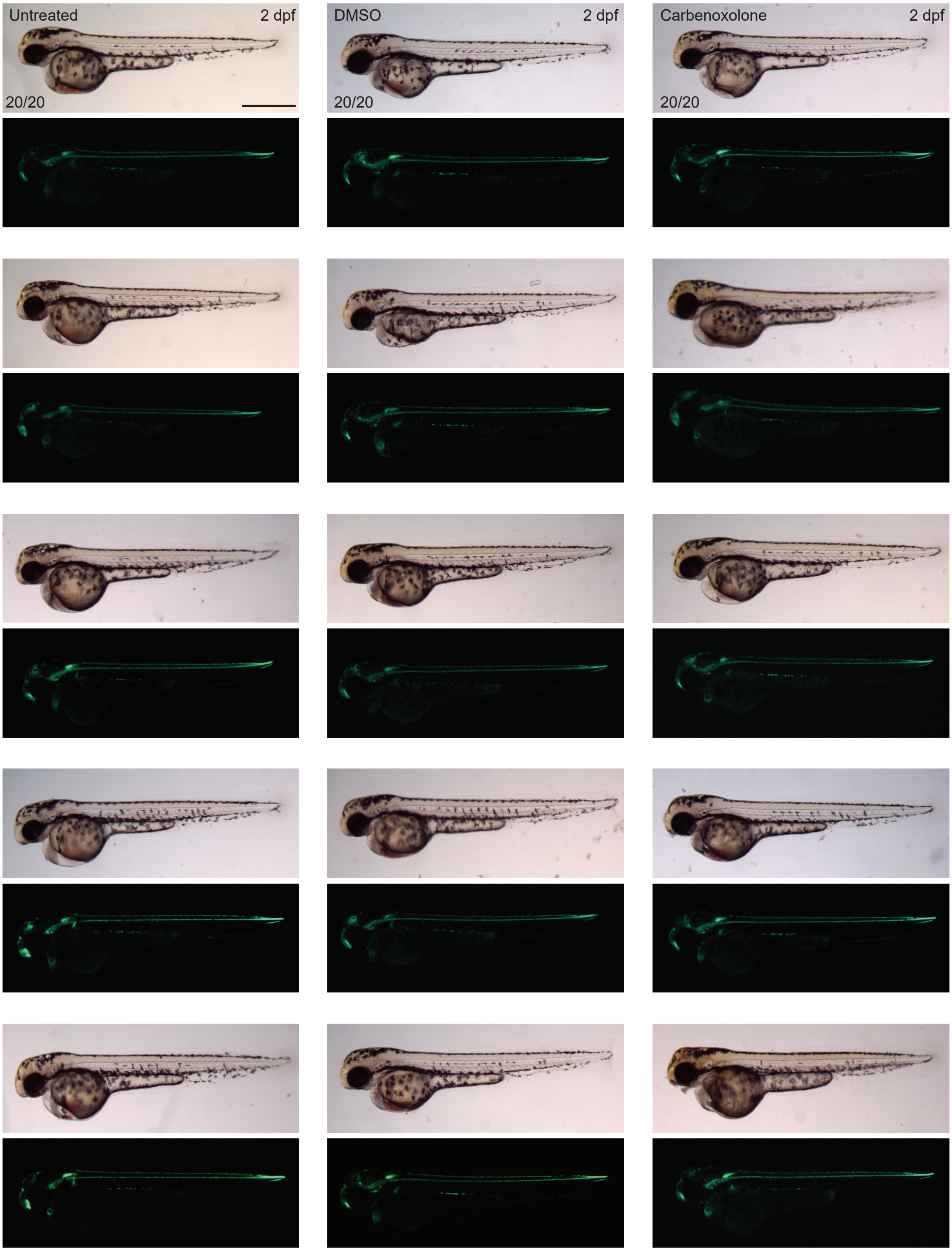

Supplementary Figure 15. Carbenoxolone is not toxic to ECs. *Tg(foxj1a:egfp)* embryos were left untreated or treated with DMSO (vehicle control) or carbenoxolone (1 μM) at 18-48 hpf and then imaged under a fluorescence stereomicroscope. Lateral view anterior to the left. Scale bar = 650 μm.

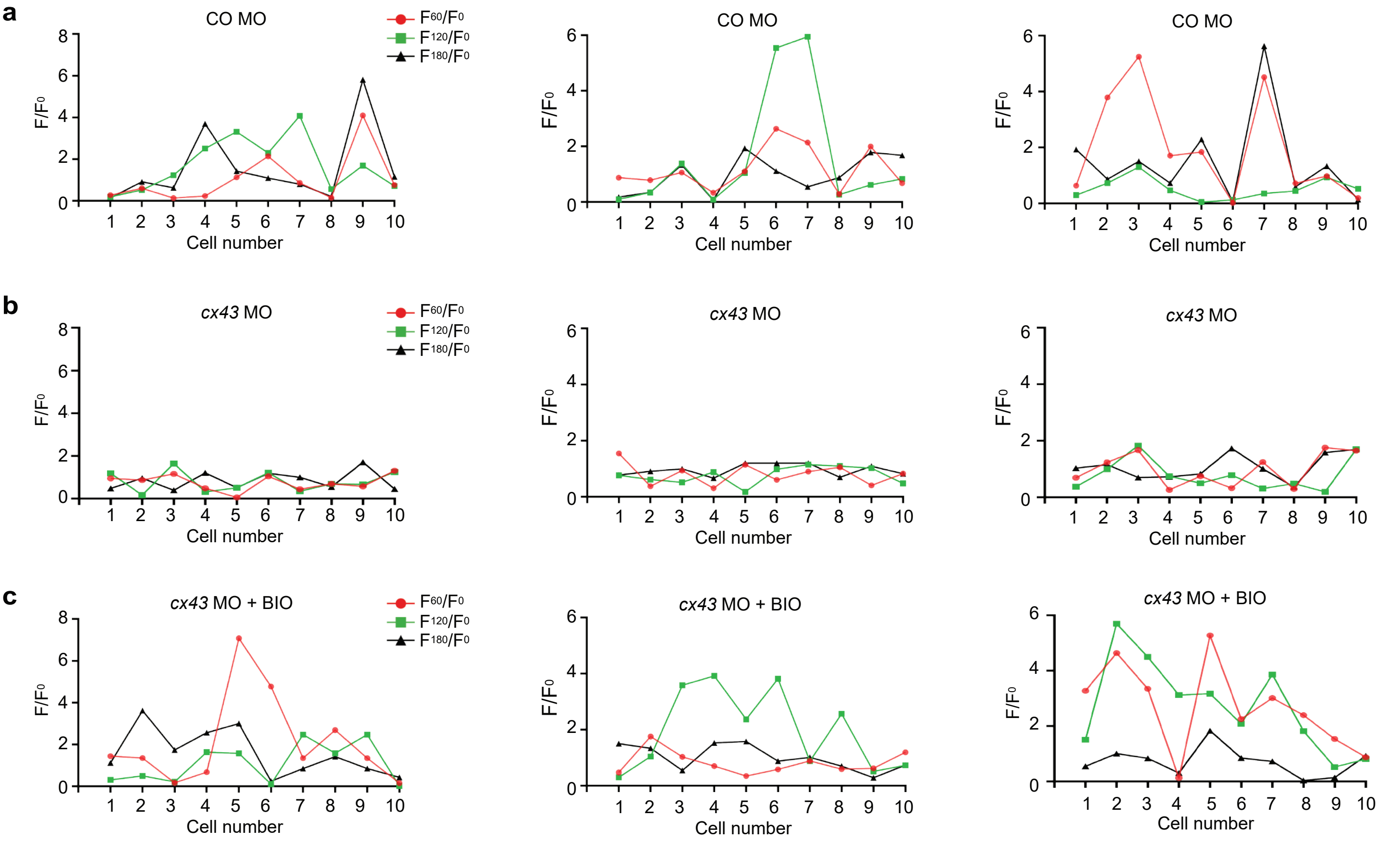

Supplementary Figure 16. Cx43 gap junctions play a role in Ca^{2+} propagation among spinal ECs. (**a-c**) *Tg(foxj1a:GCaMP6s)* embryos expressing a calcium indicator (GCaMP6s) in ECs were microinjected at one-cell stage with control (CO) MO (**a**) or *cx43* MO, and *cx43* morphants were left untreated (**b**) or treated with BIO (5 μM) at 12-48 hpf (**c**). Subsequently, they were subjected to time-lapse imaging for 3 min (20 frames/min) with a confocal microscope. The GFP fluorescence intensity in ten cells (1-10) in each embryo was individually assessed at 0 (F_0), 60 (F_{60}), 120 (F_{120}) and 180 sec (F_{180}), and presented as F_{60}/F_0, F_{120}/F_0 and F_{180}/F_0.

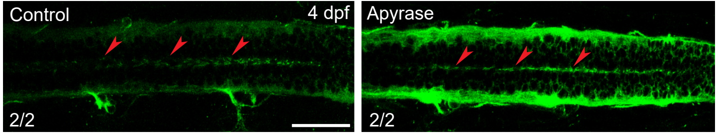

Supplementary Figure 17. Cx43 hemichannels are not implicated in the maintenance of ependymal motile cilia. Apyrase (0.175 U) was microinjected into the hindbrain ventricles of zebrafish larvae at 4 dpf and the larvae were immunostained with anti-acetylated α-tubulin antibody 4 hr after injection. Arrowheads represent motile cilia. Dorsal view anterior to the left. Scale bar = 20 µm.

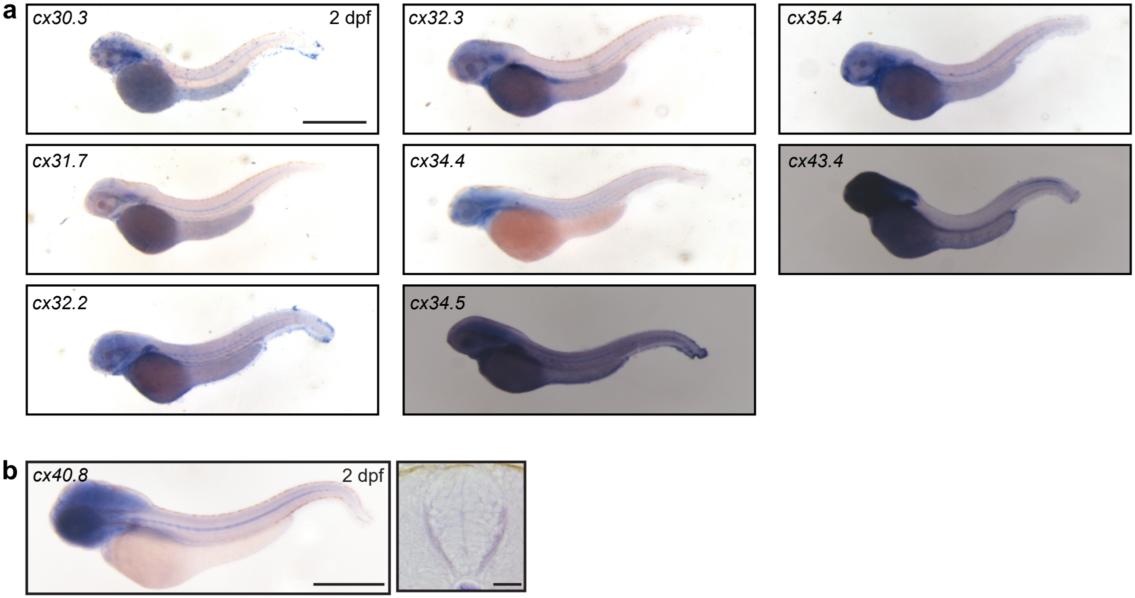

Supplementary Figure 18. Expression patterns of various *cx* genes in zebrafish embryos.
(a, b) Embryos at 2 dpf were probed with the indicated *cx* riboprobes. Lateral view anterior to the left. Scale bar = 450 µm. (**b**) Right panel shows the cross-sectioned image of the SC ventral to the bottom. Scale bars: lateral view = 650 µm; cross-section = 20 µm.

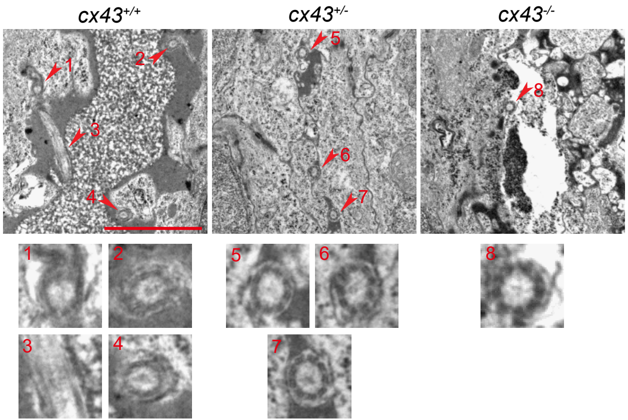

Supplementary Figure 19. A decrease in ependymal motile cilia in *cx43*$^{-/-}$ embryos compared to WT siblings. Larvae at 10 dpf from mating of *cx43*$^{+/-}$ zebrafish were cut into cranial and caudal halves. The cranial half was used for *cx43* genotyping, and the caudal half was coronally sectioned and then processed for TEM. Arrowheads represent motile cilia. Regions marked by arrowheads are magnified in the respective lower panels. Scale bar = 2 μm.

Supplementary Table 1. Sequences of primers (5’ → 3’) used to construct indicated plasmids.

Genes	Sequences
cx30.3	Forward: TAGTCATCGATACCATGAGTTGGGGAGCACT<br>Reverse: GACTATCTAGATTAAACAGTCTTATT
cx31.7	Forward: TAGTCATCGATACCATGAATTGGGCATCCTT<br>Reverse: GACTACTCGAGCTAGAAAGCTGAGCA
cx32.2	Forward: TAGTCATCGATACCATGGGAGACTGGGGGTT<br>Reverse: GACTATCTAGATTA AGCCTCAGGTTT
cx32.3	Forward: TAGTCATCGATACCATGGGAGACTGGGGATT<br>Reverse: GACTATCTAGACTAGTGATCTTCCAT
cx34.4	Forward: TAGTCATCGATACCATGAATTGGGCTTTTCT<br>Reverse: GACTACTCGAGTCAGTCACGTTCGCA
cx34.5	Forward: TAGTCATCGATACCATGGGCGAGTGGGATTT<br>Reverse: GACTACTCGAGTCAGATATGAACCTC
cx35.4	Forward: TAGTCATCGATACCATGGACTGGAAGACTTT<br>Reverse: GACTATCTAGATTAGATGATACCAGA
cx40.8	Forward: CGGCCTAAGATCTGGACCATGGGTGACTGGAGCGCACT<br>Reverse: GGCTCGAGAGGCCTTGAATTCTAGATGTCAAGATCATCC
cx43	Forward: TGCTGGGATCCATCTCCAATTCACATGCACAAG<br>Reverse: TGCTCCTCGAGTCATACCACCACCCAAAATACA
wnt4b	Forward: ACTAATATCGATACCATGCCAACAGTCTCCTCTGTG<br>Reverse: ACTATCCTCGAGTTATTCTCGGCAGGTGTGTA
wnt11	Forward: ACTAATATCGATACCATGAAGCGAACCTTCCCTTC<br>Reverse: ACTATCCTCGAGTCATTTGCAGACGTATTTCTC
plcβ1	Forward: TAGTCGATATCACCCATGAAGTGGGATGATGACTGC<br>Reverse: GACTACTCGAGCTCTCCACTCTCCGTCTGTC
plcβ4	Forward: TAGTCGGATCCACCGAGCTGGATTGCTGGGATGG<br>Reverse: GACTACTCGAGCTTCCACATACGTGCCGATC
plcδ1a	Forward: TACGTATCGATACCATGTCTTGTCCATTTAAACCTCTC<br>Reverse: GACTACTCGAGTTAAGCATCTAGAAGCAT
plcδ3a	Forward: TAGTCGGATCCACCATGCTGGGGAGAAAGAAG<br>Reverse: GACTAGAATTCTCAGGCCTTGCCTATCCTCTC
plcδ3b	Forward: TAGTCGGATCCACCATGTTGAGAAAGAAGAAGACTG<br>Reverse: GACTACTCGAGTTATTCTCGGGCACTATGTG
plcδ4a	Forward: TAGTCGGATCCACCATGACAGAACGACAGATGGCTTC<br>Reverse: GACCGGAATTCTCAGGTTAAGTTTGTAATC
plcδ4b	Forward: TAGTCATCGATACCATGGAATCACCTCAAGCATGCC<br>Reverse: GACCGTCTAGATTAAGCATGGACATTAGAACTGTG
plch2a	Forward: TAGTCGGATCCACCGGGACATCTTCAACCCTG<br>Reverse: GACTACTCGAGCTTGATCAGTATTTTGCCTT
plcl2	Forward: TAGTCGGATCCACCCATTTACACAGGCCACACAATGAC

	Reverse: GACTACTCGAGATGATAGCCGGACGGAGTAC
plcxd1	Forward: TAGTCGGATCCATGACATCGGAGGTCAGTCAAG<br>Reverse: GACTACTCGAGTCAAGGTTGTGGTAAAATGGTC
plcxd2	Forward: TAGTCGGATCCACCATGAAGACGCGACCCACAG<br>Reverse: GACTACTCGAGTCATTTGGTATCCCGATC
Cx43 T154A	Forward: GGTGGCCTGCTGAGAgCCTACATCATCAGC<br>Reverse: GCTGATGATGTAGGcTCTCAGCAGGCCACC
Cx43 Δ257-382 I	Forward: GAATTCCCGGGATATCGTCGAC<br>Reverse: TGGGCTCAGTGGGCCGGT
Cx43 Δ257-382 II	Forward: TAAACAGGCTTGAACATCAAGCTGCCAATC<br>Reverse: AGCTGGGTACCGGGCCCAATG
GCaMP6s	Forward: GGGGACAAGTTTGTACAAAAAAGCAGGCTCCATGGGTTCTCATCATCATC<br>Reverse: GGGGACCACTTTGTACAAGAAAGCTGGGTGTCACTTCGCTGTCATCATTTG

Lower case indicates mutated nucleotides.

Supplementary Table 2. Sequences of primers (5'→ 3') used to construct plasmids harboring WT *plcδ3a* promoter or *plcδ3a* promoter lacking Tcf binding elements (TBEs).

Genes	Sequences
plcδ3a promoter	Forward: CGCGCATGTCCGTTCAACTTGAATATTC<br>Reverse: GAATATTCAAGTTGAACGGACATGCGCG
Deletion of the 1st TBE	Forward: ATTACCACGCGCATGTCCGTTTGAATATTCATATCCGCCC<br>Reverse: GGGCGGATATGAATATTCAAACGGACATGCGCGTGGTAAT
Deletion of the 2nd and 3rd TBEs	Forward: CTACCTTTTTGTTCAAGATAATTCAAGCACCTTGGAATTAAGTG<br>Reverse: CACTTAATTCCAAGGTGCTTGAATTATCTTGAACAAAAAGGTAG

Supplementary Table 3. Sequences of primers (5’ → 3’) used for qPCR.

Genes	Sequences
18S rRNA	Forward: AGGGACAAGTGGCGTTCAGC<br>Reverse: GCAGGGTAGGCACACGTTGA
actb1	Forward: ATTGTTGGACGACCCAGACA<br>Reverse: CGGAGCTCATTGTAGAAGGTGT
cx40.8	Forward: ATGAGGCAGCTTTCGAAGGAA<br>Reverse: GACGACCAACCTTTAAGCTGGA
cx43	Forward: CAAGTCCATCTGTGAGGTGGTC<br>Reverse: GATTCGTTTGAAGAGCACGTAG
foxj1a	Forward: AGGAAGGACAGGATTTGTCAGG<br>Reverse: GGCTGTCGTCTAAATTGTCGG
gapdh	Forward: GTGGAGTCTACTGGTGTCTTC<br>Reverse: GTGCAGGAGGCATTGCTTACA
plcδ3a	Forward: GGTGGAAGATCATGACCACAA<br>Reverse: CCGTACCACCTTGACATGGA
rfx2	Forward: ACAAGGCTGAGGAGAAACGA<br>Reverse: ACCGGTGGCCTGAACTAAAA
rfx3	Forward: CACTCACAGCATGGCCAACAA<br>Reverse: CAATCGCCATTTCAATCGTAGCAG
