# Supplementary material for: Wnt-PLC-IP3-Connexin-Ca2+ axis maintains ependymal motile cilia in zebrafish spinal cord
Source: Nat Commun. 2020 Apr 20;11:1860. doi: 10.1038/s41467-020-15248-2 (PMC7170879; doi:10.1038/s41467-020-15248-2)
Supplement: Supplementary file 2 — Description of Additional Supplementary Files [file 41467_2020_15248_MOESM2_ESM.pdf]

Description of Additional Supplementary Files

Supplementary Movie 1. *Tg(bactin2:Arl13b-GFP)* embryos expressing GFP in motile cilia were microinjected at one-cell stage with control MO and subjected to time-lapse imaging for 2 min (67 frames/sec) with a multiphoton confocal microscope. Dorsal view anterior to the left. Scale bar = 7.5 µm.

Supplementary Movie 2. *Tg(bactin2:Arl13b-GFP)* embryos expressing GFP in motile cilia were microinjected at one-cell stage with *cx43* MO and subjected to time-lapse imaging for 2 min (67 frames/sec) with a multiphoton confocal microscope. Dorsal view anterior to the left. Scale bar = 7.5 µm.

Supplementary Movie 3. *Tg(foxj1a:GCaMP6s)* embryos expressing a calcium indicator (GCaMP6s) in ECs were microinjected at one-cell stage with control MO and subjected to time-lapse imaging for 3 min (20 frames/min) with a confocal microscope. Dorsal view anterior to the left. Scale bar = 20 µm.

Supplementary Movie 4. *Tg(foxj1a:GCaMP6s)* embryos expressing a calcium indicator (GCaMP6s) in ECs were microinjected at one-cell stage with *cx43* MO and subjected to time-lapse imaging for 3 min (20 frames/min) with a confocal microscope. Dorsal view anterior to the left. Scale bar = 20 µm.
