# Supplementary material for: Wnt-PLC-IP3-Connexin-Ca2+ axis maintains ependymal motile cilia in zebrafish spinal cord
Source: Nat Commun. 2020 Apr 20;11:1860. doi: 10.1038/s41467-020-15248-2 (PMC7170879; doi:10.1038/s41467-020-15248-2)
Supplement: Supplementary file 7 — Reporting Summary [file 41467_2020_15248_MOESM7_ESM.pdf]

nature research

Corresponding author(s): Seok-Yong Choi

Last updated by author(s): Feb 21, 2020

Reporting Summary

Nature Research wishes to improve the reproducibility of the work that we publish. This form provides structure for consistency and transparency in reporting. For further information on Nature Research policies, see Authors & Referees and the Editorial Policy Checklist.

Statistics

For all statistical analyses, confirm that the following items are present in the figure legend, table legend, main text, or Methods section.

n/a	Confirmed	
☐	☒	The exact sample size (*n*) for each experimental group/condition, given as a discrete number and unit of measurement
☐	☒	A statement on whether measurements were taken from distinct samples or whether the same sample was measured repeatedly
☐	☒	The statistical test(s) used AND whether they are one- or two-sided<br>*Only common tests should be described solely by name; describe more complex techniques in the Methods section.*
☒	☐	A description of all covariates tested
☒	☐	A description of any assumptions or corrections, such as tests of normality and adjustment for multiple comparisons
☐	☒	A full description of the statistical parameters including central tendency (e.g. means) or other basic estimates (e.g. regression coefficient) AND variation (e.g. standard deviation) or associated estimates of uncertainty (e.g. confidence intervals)
☐	☒	For null hypothesis testing, the test statistic (e.g. *F*, *t*, *r*) with confidence intervals, effect sizes, degrees of freedom and *P* value noted<br>*Give P values as exact values whenever suitable.*
☒	☐	For Bayesian analysis, information on the choice of priors and Markov chain Monte Carlo settings
☒	☐	For hierarchical and complex designs, identification of the appropriate level for tests and full reporting of outcomes
☒	☐	Estimates of effect sizes (e.g. Cohen's *d*, Pearson's *r*), indicating how they were calculated

Our web collection on statistics for biologists contains articles on many of the points above.

Software and code

Policy information about availability of computer code

Data collection	Zen Black (version 8.1), LAS X, DigitalMicrograph (version 3.0)
Data analysis	Microsoft Excel (version 2013), GraphPad Prism (version 5.0), ImageJ (version 1.51), PhotoShop (version CS6), ZiFiT Targeter (version 4.2)

For manuscripts utilizing custom algorithms or software that are central to the research but not yet described in published literature, software must be made available to editors/reviewers. We strongly encourage code deposition in a community repository (e.g. GitHub). See the Nature Research guidelines for submitting code & software for further information.

Data

Policy information about availability of data

All manuscripts must include a data availability statement. This statement should provide the following information, where applicable:

- Accession codes, unique identifiers, or web links for publicly available datasets
- A list of figures that have associated raw data
- A description of any restrictions on data availability

The authors declare that the data supporting the findings of this study are available within the paper and its supplementary information files. Reagents are available upon request.

Field-specific reporting

Please select the one below that is the best fit for your research. If you are not sure, read the appropriate sections before making your selection.

☒ Life sciences ☐ Behavioural & social sciences ☐ Ecological, evolutionary & environmental sciences

Life sciences study design

All studies must disclose on these points even when the disclosure is negative.

Sample size	Sample sizes were chosen based on clutch sizes of embryos per used model organism. Injection-based assays and stainings were performed to reach effect sizes of >10 of the observed effect in all combined experiments of one type.
Data exclusions	For morpholino-injection experiments, embryos with abnormal morphology were excluded for subsequently experiments. The exclusion criteria were pre-established. In other experiments, no data were excluded.
Replication	The exact number of replication for all experiments was described in figure legends and our attempts at replication were successful.
Randomization	For zebrafish experiments, embryos with different genotype were selected randomly and then subjected to either microinjection or drug treatment. Subsequently, they were selected randomly, processed for immunostaining, and then subjected to confocal microscopy. For mice experiment, mice were allocated randomly.
Blinding	The investigators were blinded to the animal genotype, drug treatment and data analysis.

Reporting for specific materials, systems and methods

We require information from authors about some types of materials, experimental systems and methods used in many studies. Here, indicate whether each material, system or method listed is relevant to your study. If you are not sure if a list item applies to your research, read the appropriate section before selecting a response.

Materials & experimental systems

n/a	Involved in the study	
☐	☒	Antibodies
☐	☒	Eukaryotic cell lines
☒	☐	Palaeontology
☐	☒	Animals and other organisms
☐	☒	Human research participants
☒	☐	Clinical data

Methods

n/a	Involved in the study	
☒	☐	ChIP-seq
☒	☐	Flow cytometry
☒	☐	MRI-based neuroimaging

Antibodies

Antibodies used

1. Anti-GFAP antibody: GeneTex, GTX128741.
2. Anti-acetylated a-tubulin antibody: Sigma, T6793, 6-11B-1.
3. Anti-γ-tubulin antibody: Sigma, T6557.
4. Alexa Fluor 488-conjugated secondary antibody: Life Technologies, R37120.
5. Alexa Fluor 555-conjugated secondary antibodies: Life Technologies, A32732.
6. Anti-CX43 antibody: Life Technologies, 13-8300.
7. Anti-Phospho-Cx 43 (Ser368) antibody: Cell Signaling Technology, 3511.
8. Anti-ARL13B antibody: A gift from Dr. Hyuk Wan Ko.

Validation

1. Anti-GFAP antibody: Host: rabbit; clonality: polyclonal; Application: WB, IHC; Reactivity: zebrafish (https://www.genetex.com/Product/Detail/Gfap-antibody/GTX128741).
2. Anti-acetylated a-tubulin antibody: Host: mouse; Application: WB, IHC; Reactivity: mouse, rat, chicken, protista, bovine, invertebrates, human, monkey, pig, frog, plant, hamster (https://www.sigmaaldrich.com/catalog/product/sigma/t6793?lang=ko&region=KR)
3. Anti-acetylated γ-tubulin antibody: Host: mouse; Application: WB, IHC; Reactivity: bovine, mouse, rat, chicken, Xenopus, human, hamster, canine (https://www.sigmaaldrich.com/catalog/product/sigma/t6557?lang=ko&region=KR)
4. Alexa Fluor 488-conjugated secondary antibody: https://www.thermofisher.com/search/results?query=37120&focusarea=%EC%A0%84%EC%B2%B4%EA%B2%80%EC%83%89
5. Alexa Fluor 555-conjugated secondary antibodies: https://www.thermofisher.com/antibody/product/Goat-anti-Rabbit-IgG-H-L-Highly-Cross-Adsorbed-Secondary-Antibody-Polyclonal/A32732
6. Anti-CX43 antibody: Host: mouse; Application: WB, IHC, IF and ELISA; Reactivity: canine, human, mouse, rat (https://www.thermofisher.com/antibody/product/Connexin-43-Antibody-clone-CX-1B1-Monoclonal/13-8300)
7. Anti-Phospho-Cx 43 (Ser368) antibody: Host: rabbit; Application: WB; Reactivity: canine, human, mouse, rat, hamster and monkey (https://www.cellsignal.com/products/primary-antibodies/phospho-connexin-43-ser368-antibody/3511)
8. Anti-ARL13B antibody: Kim, Y.K., Kim, J.H., Yu, Y.S., Ko, H.W. & Kim, J.H. Localization of primary cilia in mouse retina. Acta Histochem 115, 789-794 (2013).

Eukaryotic cell lines

Policy information about cell lines

Cell line source(s)	HEK cell line from ATCC
Authentication	We directly purchased the HEK cell line from ATCC and had not authenticated it since then.
Mycoplasma contamination	The HEK cell line was tested negative for mycoplasma contamination.
Commonly misidentified lines (See ICLAC register)	No commonly misidentified cell lines were used in the study

Animals and other organisms

Policy information about studies involving animals; ARRIVE guidelines recommended for reporting animal research

Laboratory animals	Zebrafish (AB strain) were obtained from the Zebrafish International Resource Center. Sex is not determined at the developmental stages of zebrafish embryos and larvae used in this study. Cx43 knockout mice pup (postnatal day 1) were used in this study and were not selected for gender. Gender of the mice used in this study was not recorded before experiments. Mice were maintained in an enclosed, specific pathogen-free (SPF) facility.
Wild animals	This study did not use wild-caught animals for data collection.
Field-collected samples	This study did not use field-collected samples for data collection.
Ethics oversight	Zebrafish experiments were approved by the Chonnam National University Medical School Institutional Animal Care and Use Committee (IACUC; project number: 2017-7). Mouse studies were approved by the Asan Institute for Life Sciences IACUC (project number: 2015-12-158). All animal experiments were conducted in accordance with relevant guidelines and regulations of the Republic of Korea.

Note that full information on the approval of the study protocol must also be provided in the manuscript.

Human research participants

Policy information about studies involving human research participants

Population characteristics	*Describe the covariate-relevant population characteristics of the human research participants (e.g. age, gender, genotypic information, past and current diagnosis and treatment categories). If you filled out the behavioural & social sciences study design questions and have nothing to add here, write "See above."*
Recruitment	*Describe how participants were recruited. Outline any potential self-selection bias or other biases that may be present and how these are likely to impact results.*
Ethics oversight	Written informed consents for the use of tissue from the deceased were obtained from the family of the deceased.

Note that full information on the approval of the study protocol must also be provided in the manuscript.
